# Synthetic strategies for aryl/heterocyclic selenides and tellurides under transition-metal-catalyst free conditions

**DOI:** 10.1039/d0ra10629a

**Published:** 2021-02-10

**Authors:** Debasish Kundu

**Affiliations:** Department of Chemistry, Government General Degree College at Mangalkote (Affiliated to The University of Burdwan) Khudrun, Purba Bardhaman 713143 India chem.debasishkundu@mangalkotegovtcollege.org debiitkgp123@gmail.com

## Abstract

Aryl and heteroaryl selenides and tellurides are found to have broad applications in the diverse fields such as medicine, biology, materials science, pharmaceutical *etc.* and thus their synthesis remains a challenging field for synthetic chemists in last decade. Although a large no of methodologies have been developed based on metal catalyzed C–Se/Te coupling, a large number of researches has been focused on developing metal catalyst free protocols due to their sustainability in recent times. This review covers all the recent developments in last decade on their synthesis under metal catalyst free conditions by using different sustainable techniques *e.g.* greener reagents and solvents, ball milling, visible light photocatalysis, microwave, ultrasound *etc.*

## Abstract 2

Synthesis of aryl and heteroaryl selenides and tellurides are summerized under transition free sustainable conditions by using greener reagents and techniques such as ball milling, microwave heating, ultrasound, visible light photocatalysis *etc.*

## Graphical abstract

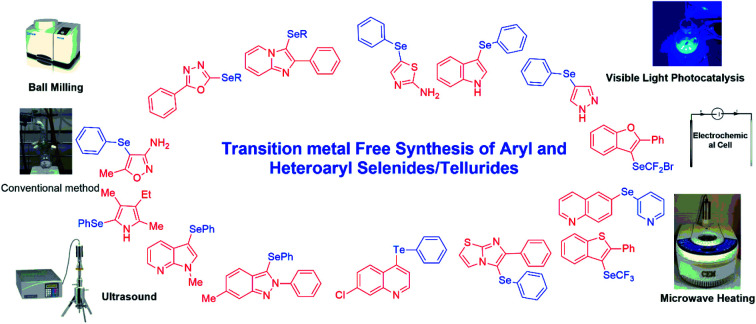

## 1 Introduction

**Fig. 1:**
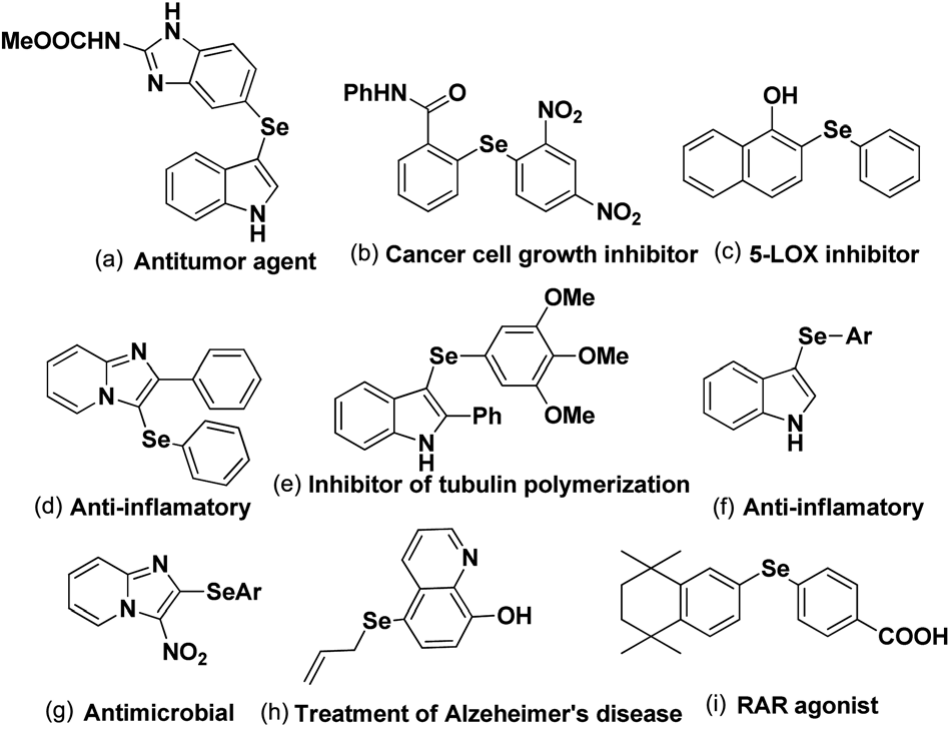
Examples of biologically important aryl/heteroaryl selenides.

C–Se/C–Te bond formations for the synthesis of organoselenides and tellurides are getting much attention from organic chemists due to their important applications in biological, environmental and pharmaceutical fields of study.^1^ They also have great significance in structural chemistry,^2^ materials science^3^ and in synthetic chemistry acting as reagents in broad array of synthesis and catalysis.^4^ Se and Te based molecules were also found to have interesting applications in semiconductors, magnets and NLO materials.^5^ Furthermore the increasing interests in selenium and tellurium chemistry is coming from the recent developments of Se- and Te-based organocatalysts which were found effective in several functional group transformations under sustainable condition for the synthesis of bio-active molecules.^6^ Although organotellurides are less explored, organoselenides which are less toxic than selenium, were found to have diverse applications in medicinal and biological fields by showing anticancer, anti-HIV and anti-bacterial activities.^7^ Aryl and heteroaryl selenides are found to have large array of applications against several human diseases and thus been applied in human body as potential therapy against them (Fig. 1).^8,9^ Among heteroaryl selenides *N*-based heteroaryl selenides such as selenylindoles, selenylimidazo[1,2-*a*]pyridines were found most potential against human diseases due to the biological importance of N-heterocycles.^10–12^ Thus developing synthetic methodologies of C–Se bond formations on arenes and heteroarenes has become a research hotspot in recent times. In last two decades transition metal catalyzed cross-coupling reactions have become a powerful tool for the synthesis of aryl/heteroaryl selenides.^13^ However, use of expensive and in some cases toxic metal salts, ligands, harsh conditions, high temperature *etc.* were the serious limitations of those protocols.

**Fig. 2:**
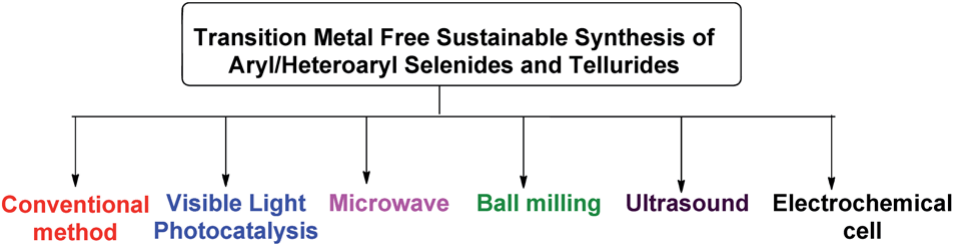
Transition metal free sustainable tools for synthesis of aryl and heteroaryl selenides.

Beletskaya and Ananikov *et al* summarized transition metal catalyzed C–S, C–Se and C–Te cross-coupling reactions.^13^ Lenardao and coworkers highlighted different non-conventional reaction media for the synthesis of organochalcogenides.^14^ Ranu and coworkers have summarized microwave assisted protocols for the synthesis of organochalcogenides.^15^ A review based on application of diselenides for the synthesis of heteroaryl selenides was reported by Arsenyan *et al.* which mostly covered transition metal catalyzed protocols along with a few metal free electrophilic selenylations.^16^ In search of developing environment friendly and economic protocols for the assembly of aryl/heteroaryl C–Se/C–Te bonds, a number of transition metal catalyst free protocols have been developed in last decade for the synthesis of aryl and heteroaryl selenides under sustainable conditions. To the best of our knowledge no exclusive review is present till date in this emerging field which covered all the well explored methodologies with broad substrate scopes for the synthesis of aryl and heterocyclic selenides and tellurides under metal catalyst free conditions. Thus here in, we have highlighted the recent advances of transition metal catalyst free strategies for the C–Se and C–Te bond formations in last decade towards the synthesis of aryl/heteroaryl selenides and tellurides by using several techniques such as conventional methods using greener solvents and reagents, microwave irradiation, ultrasound, ball-milling, visible light medium and electrochemical cell (Fig. 2).

## 2 Under conventional method

**Scheme 1:**
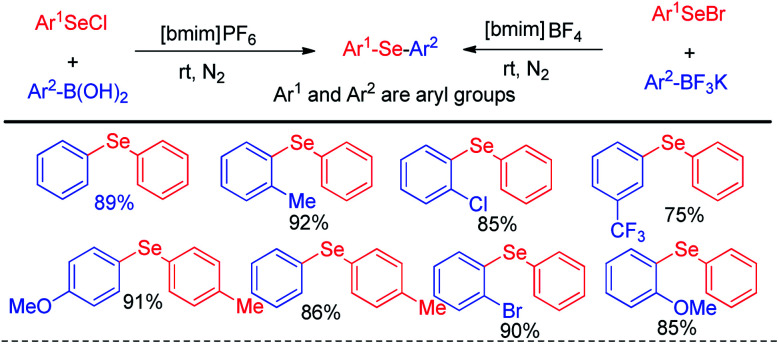
Ionic liquid mediated synthesis of diaryl selenides from boronic acids and trifluoroborates.

In the context of green chemistry, ionic liquids have appeared as a powerful solvent in last two decades due to their high boiling point, easy separation and recyclability.^17^ Lenardao and Alves *et al.* in 2011 reported ionic liquid mediated reaction of aryl selenium chlorides/bromides with aryl boronic acids for the synthesis of large array of diaryl selenides under room temperature (Scheme 1).^17^ The authors also explored the reaction with aryl trifluoroborates, however the yield was lower in comparison to the reactions with aryl boronic acids. The authors have managed to recycle the ionic liquid solvent for five times without any appreciable loss of yield.

**Scheme 2:**
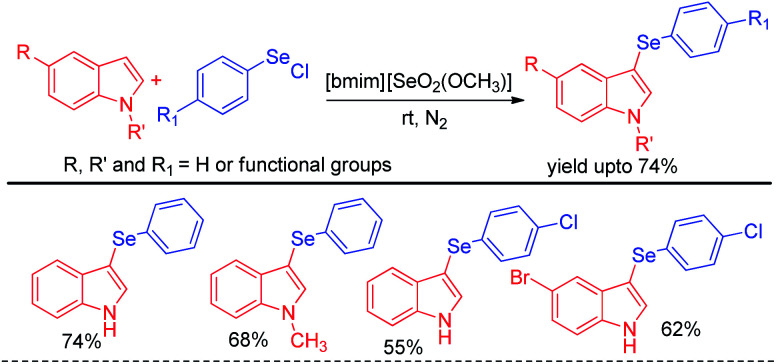
3-Organoselenylation of indoles in ionic liquid.

The idea of electrophilic selenylation by using aryl selenium halides as electrophiles was further nicely applied by the same group towards 3-organoselenylation of indoles (Scheme 2).^18^ The authors have used selenium based ionic liquid [bmim][SeO_2_(OCH_3_)] as solvent.

**Scheme 3:**
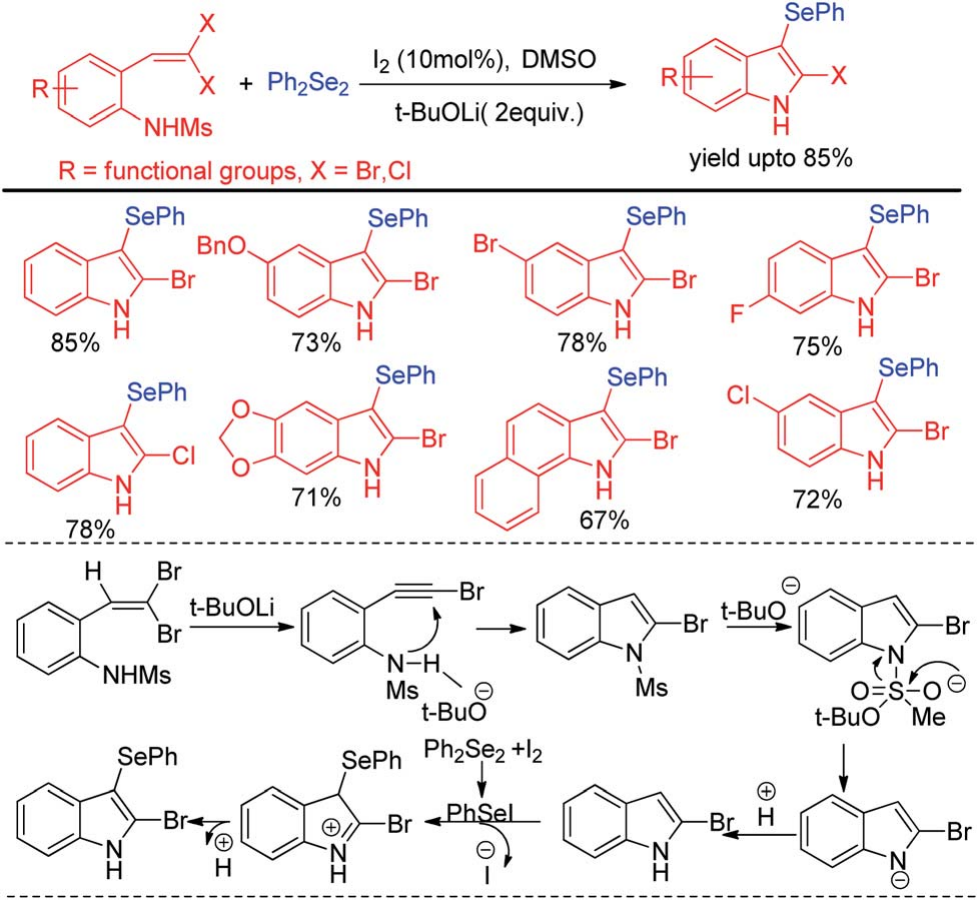
Iodine catalyzed synthesis of 2-bromo-3-selenyl-indoles by tandem reaction.

Wang and co-workers have explored synthesis of 2-bromo-3-selenyl-indoles through iodine catalysed tandem reactions of 2-(*gem*-dibromo-vinyl)-*N*-methylsulfonylanilines with diselenides by using tBuoLi in DMSO solved under 110 °C heating in sealed tube (Scheme 3).^19^ The reaction mechanism was proposed based on the synthesis of 2-bromoindole in presence of base which underwent selenylation in the electron rich 3-position with organodiselenides through the formation of active electrophile PhSeI by iodine.

**Scheme 4:**
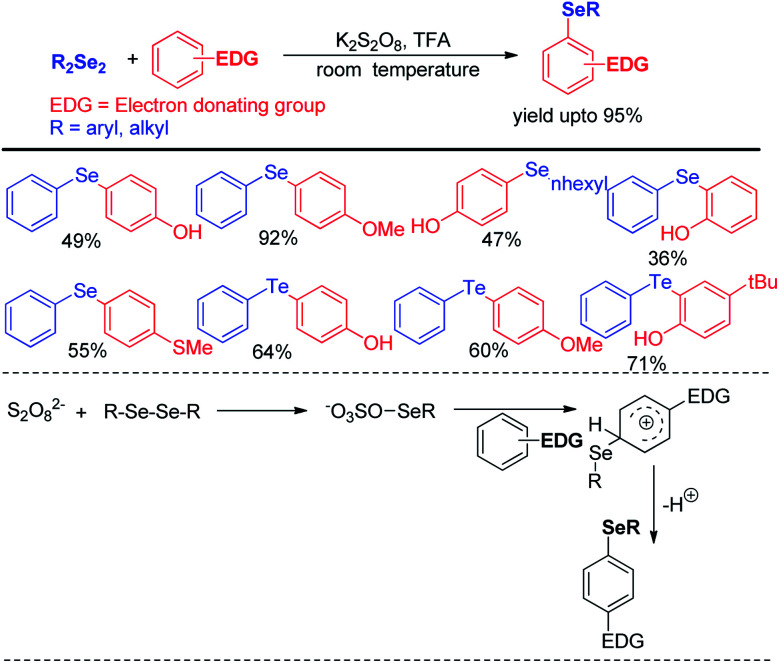
K_2_S_2_O_8_ mediated electrophilic selenylation of electron-rich arenes.

Reaction with electron rich arenes and daryldiselenides was nicely explored by Kumar and co-workers to access diarylchalogenides using K_2_S_2_O_8_ (Scheme 4).^20^

**Scheme 5:**
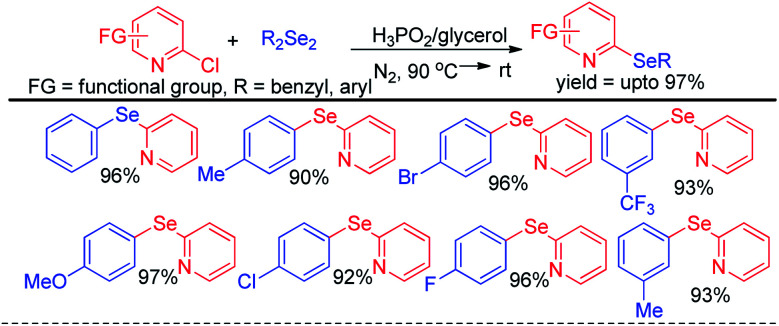
Synthesis of 2-organoselanyl pyridines by using H_3_PO_2_ as reducing agent in glycerol medium.

Arenes with different electron donating groups give moderate to high yields by electrophilic selenation at the -*ortho* or -*para* position w.r.t. the activating group in the aromatic ring. The mechanism was proposed by activation of diselenides with potassium persulfate as an oxidant to form an active intermediate which underwent electrophilic attack by electron-rich arenes to form the desired product after proton elimination. The authors also have explored the scope of this reaction for the synthesis of diarylsulphides and tellurides. Alves *et al.* has reported H_3_PO_2_ mediated synthesis of 2-organoselanyl pyridines by reacting 2-chloropyridines with organodiselenides under glycerol medium (Scheme 5).^21^ The authors successfully recycled the H_3_PO_2_/glycerol medium up to four cycles without appreciable loss of yield, however considerable loss of yield was found in 5^th^ and 6^th^ cycle. The reaction successfully synthesized a library of pyridine based selenides under sustainable conditions.

**Scheme 6:**
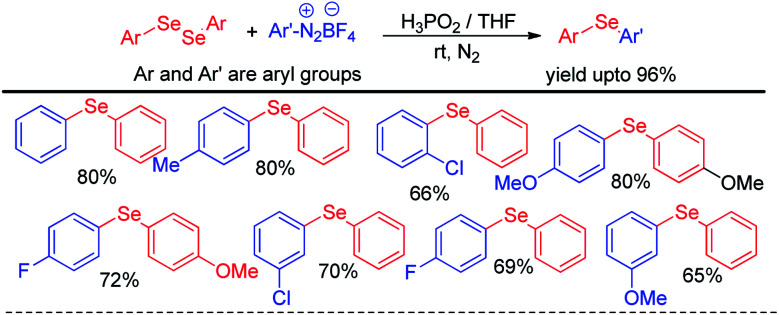
H_3_PO_2_ driven synthesis of diaryl selenides from aryl diazonium salts.

The same group later explored H_3_PO_2_ mediated simple reaction of aryl diazoniumtetrafluoroborate with diaryldiselenides for the synthesis of various diaryl selenides under room temperature in THF (Scheme 6).^22^ The authors reported *in situ* formation of phenyl selenol which acted as nucleophile and attacked aryl diazonium fluoroborate to form the product. The reaction was explored with several substituents in the aromatic ring of both diaryldiselenides and aryl diazoniumtetrafluoroborate salts.

**Scheme 7:**
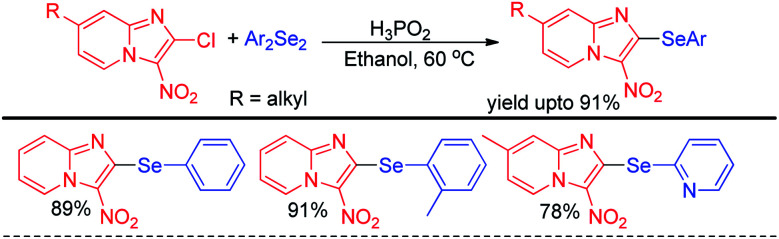
H_3_PO_2_ mediated organoselenation of imidazopyridines.

The idea of activating diselenides by H_3_PO_2_ for performing nucleophilic selenation in aromatic ring was further applied by Sharma and Bhasin *et al.* for organoselenation of electron deficient imidazopyridines (Scheme 7).^23^

**Scheme 8:**
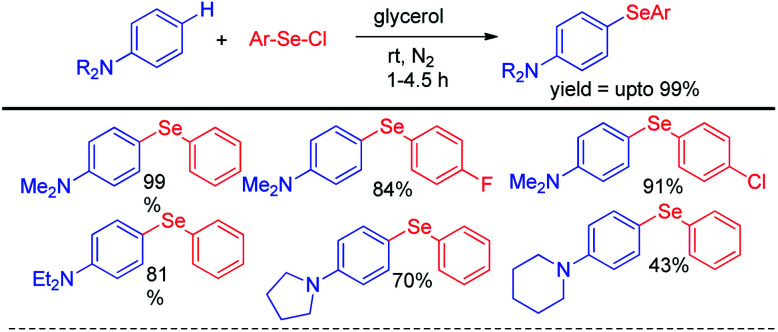
Synthesis of aryl selenyl anilines under base free conditions.

Lenardao *et al* reported C–H selenylation of aromatic tertiary amines by aryl selenyl chlorides under base free conditions in glycerol medium (Scheme 8).^24^ The reactions followed usual S_N_Ar mechanistic path. Both cyclic and acyclic amines substituted aromatic rings were found to react with aryl selenyl chlorides under the reaction conditions however yields were found lowered in case of cyclic amines substitutedarenes.

**Scheme 9:**
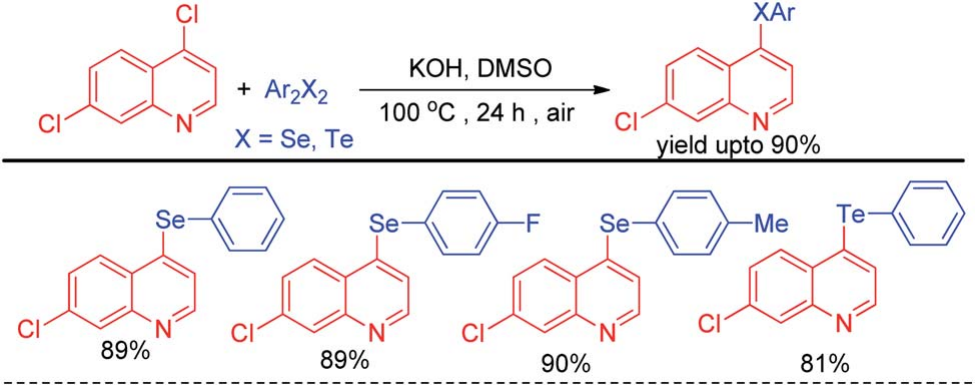
Synthesis of quinoline selenides and tellurides *via* S_N_Ar reaction.

Alves *et al.* reported synthesis of selenium and tellurium substituted quinoline derivatives by the S_N_Ar reaction of 4,7-dichloroquinoline with diselenides in presence of KOH (Scheme 9).^25^

**Scheme 10:**
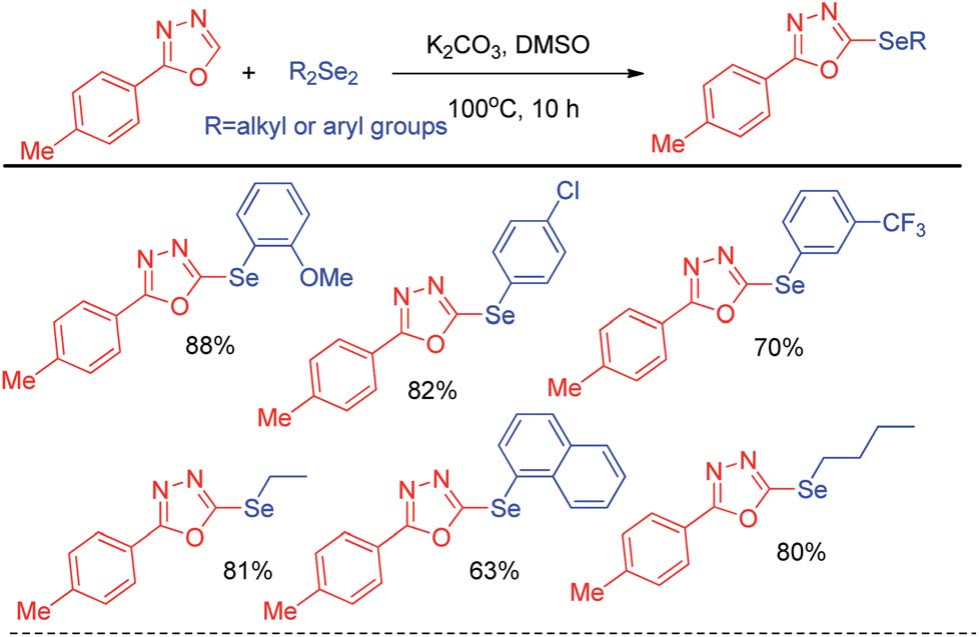
K_2_CO_3_ promoted selenylation in 1,3,4-oxadiazoles.

In 2014, Braga *et al.* have developed K_2_CO_3_ driven selenylation in 1,3,4-oxadiazoles in DMSO solvent under 100 °C heating condition (Scheme 10).^26^ The acidic hydrogen in the 1,3,4-oxadiazoles was abstracted by base and nucleophilic substitution towards diselenide resulted the desired product.

**Scheme 11:**
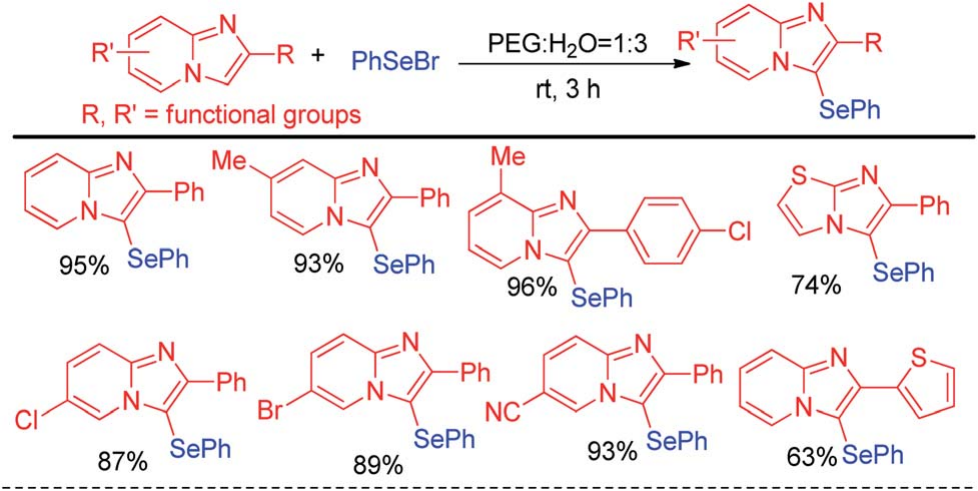
Phenyl selenylation of imidazo[1,2-*a*]pyridine and imidazo[2,1-*b*]thiazole.

Hajra and co-workers have reported synthesis of 3-phenylselenyl imidazo[2,1-*b*]pyridine by selenylation of several substituted imidazo[1,2-*a*]pyridine molecules in PEG : H_2_O solvent (1 : 3) under base free conditions in room temperature (Scheme 11).^27^ Phenyl selenyl bromide was used as electrophile and the reaction followed a nucleophilic substitution mechanism. A library of substituted imidazo[1,2-*a*]pyridine molecules bearing several substituents in the aromatic ring *e.g.* –Me, –F, –Cl, –Br, –CF_3_*etc.* underwent selenylation under this condition. The protocol was also successful for selenylation of imidazo[2,1-*b*]thiazole.

**Scheme 12:**
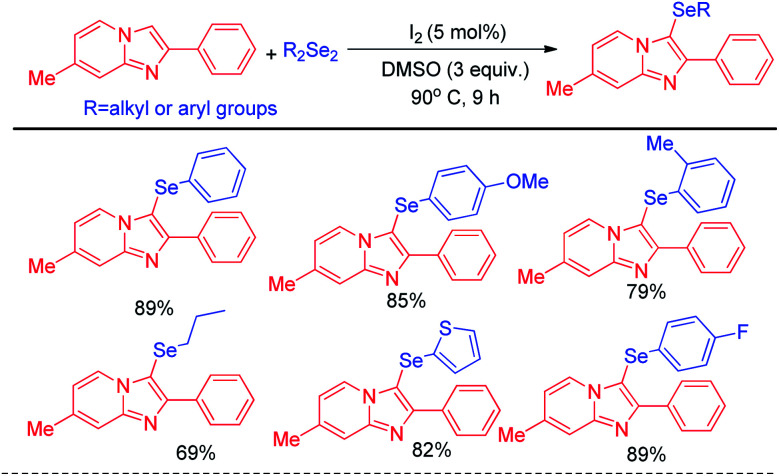
Iodine catalysed atom economical selenylation of imidazo[1,2-*a*]pyridine.

The concept of Hajra *et al.* of using organoselenium halide as electrophile in synthesizing selenolated heteroarenes was further designed atom economically by Braga and co-workers who latter reported organoselenylation in imidazo[1,2-*a*]pyridines *via* electrophilic aromatic substitution with diselenides by using catalytic amount of iodine in reaction medium which generated selenyliodide *in situ* in medium (Scheme 12).^28^ Here DMSO acted as oxidizing agent to regenerate I_2_ from iodide in reaction medium. Apart from daryldiselenides, dialkyl and diheteroaryl selenides were also successfully applied to reaction under the reaction conditions.

**Scheme 13:**
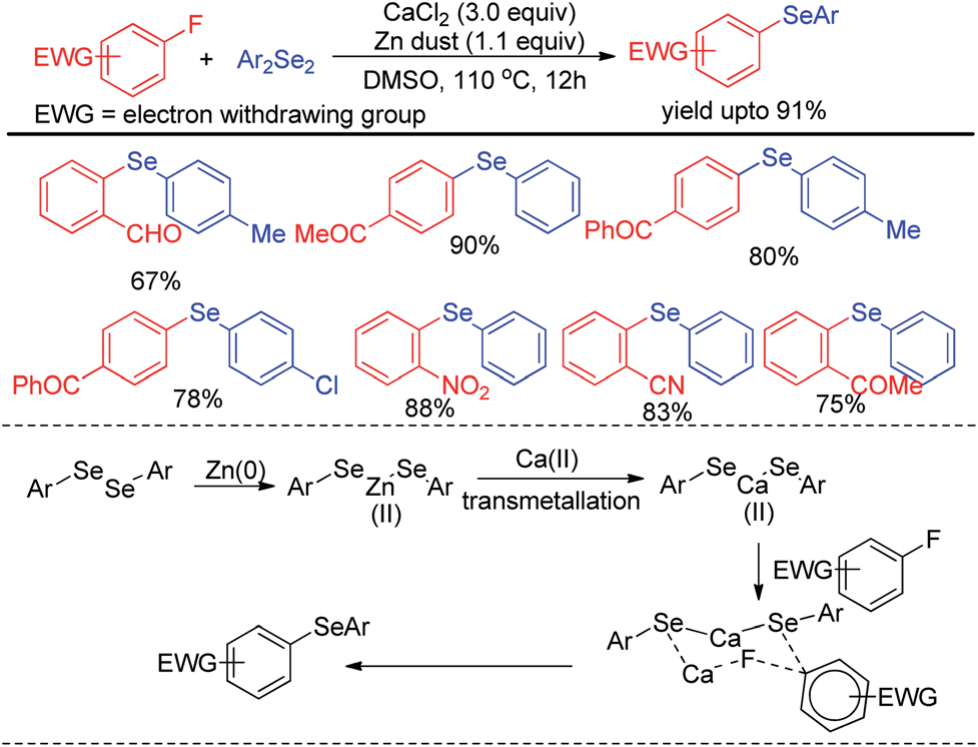
CaCl_2_ mediated selenation by C–F bond activation in fluoroarenes.

Ranu *et al.* have reported a synthesis of unsymmetrical diaryl selenides by calcium mediated aryl C–F bond substitution in activated fluoroarenes (Scheme 13).^29^ Many diaryl selenide compounds bearing several electron withdrawing substituents in the aromatic ring were synthesized with this method in high yields. The authors have proposed an interesting mechanism involving a transmetalation process of aryl selenide from Zn(ii) to Ca(ii) to produce active nucleophile Ca(SeAr)_2_. The bond was activated by Ca(ii) and thus underwent nucleophilic attack to form the product *via* six membered transition state which was established by DFT studies.

**Scheme 14:**
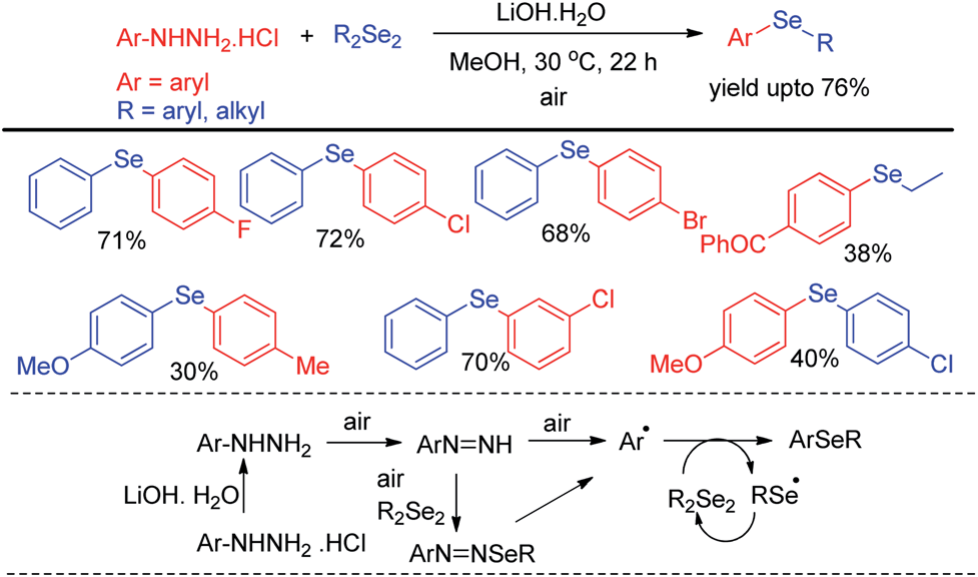
Synthesis of diaryl-selenides from aryl hydrazine hrdrochlorides under air.

Aryl hydrazine hydrochlorides were successfully applied as synthons for the synthesis of unsymmetrical diaryl selenides by Mizuno *et al. via* base promoted one pot reaction with diselenides using air as oxidant under 30 °C (Scheme 14).^30^ Several diarylselenide compounds having different substituents in the aromatic ring have been synthesized by this protocol in moderate to good yield. The authors proposed an air driven radical mechanistic approach for this reaction which underwent *via* forming aryl radical from aryl hydrazines.

**Scheme 15:**
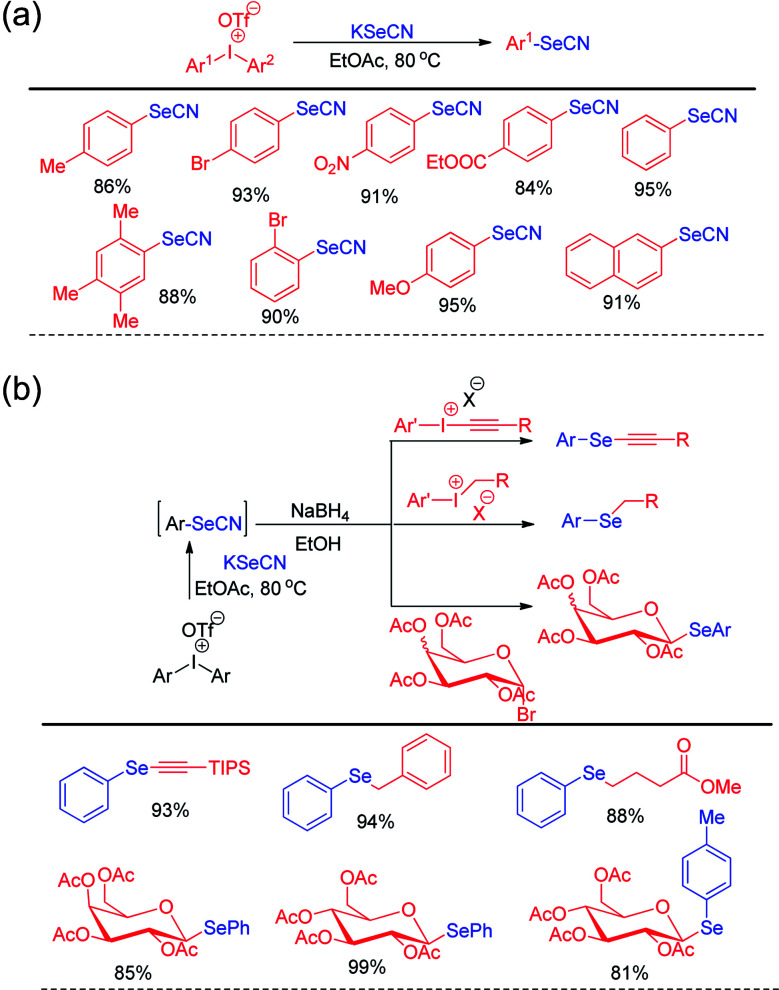
(a) Synthesis of aryl selenocyanate by reacting KSeCN with diaryliodonium salts. (b) One pot synthesis of various organoselenides *via in situ* formation of aryl selenocyante.

Steven D. Townsend and co-workers has introduced diaryl iodonium triflates for the metal free one-pot synthesis of aryl selenocyanates by reacting with KSeCN in ethyl acetate under 80 °C heating (Scheme 15a).^31^

**Scheme 16:**
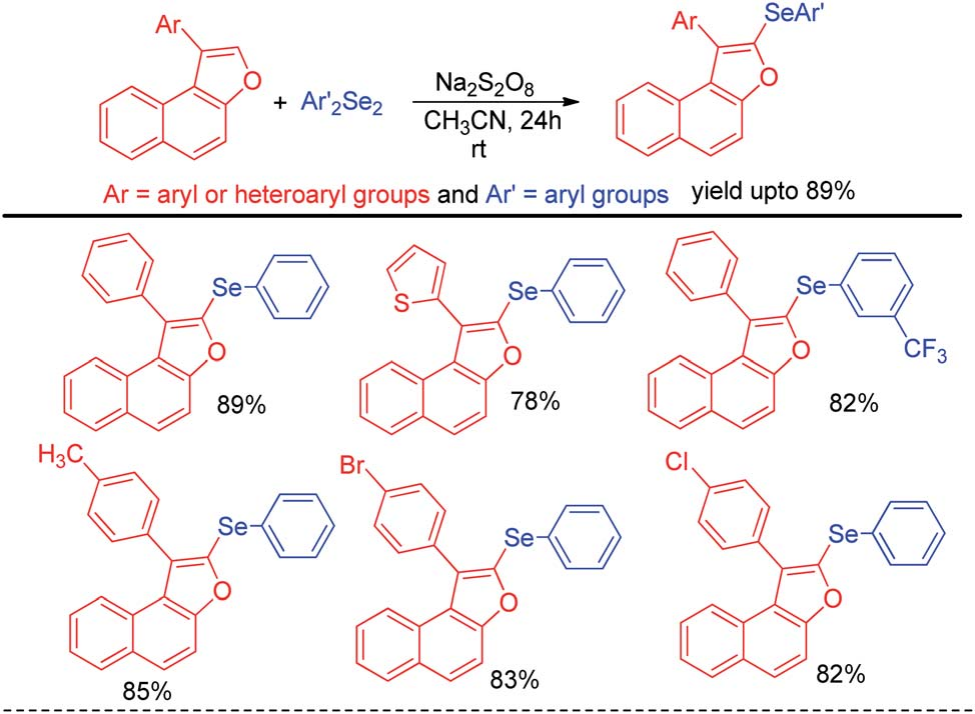
Selenylation of substituted naphthofurans under rt.

In addition to the synthesis of several aryl selenocyanates the protocol was further applied for the one pot synthesis of several important organoselenides by nucleophilic substitution in various electrophilic species such as alkynyl and benzyl iodonium salts, glycosyl bromide *etc.* (Scheme 15b). In the first step aryl selenocyanate was formed by the arylation of potassium selenocyante which was followed by reduction with NaBH_4_ and attack in electrophile. Hajra *et al.* introduced an sustainable protocol for the selenylation in furan ring of various 3-substituted naphtho[2,1-*b*]furans by reacting it with diselenides under room temperature by using sodium persulfate as oxidant (Scheme 16).^32^ However it was observed that alkyl substituted furan rings failed to initiate any conversion to the product.

**Scheme 17:**
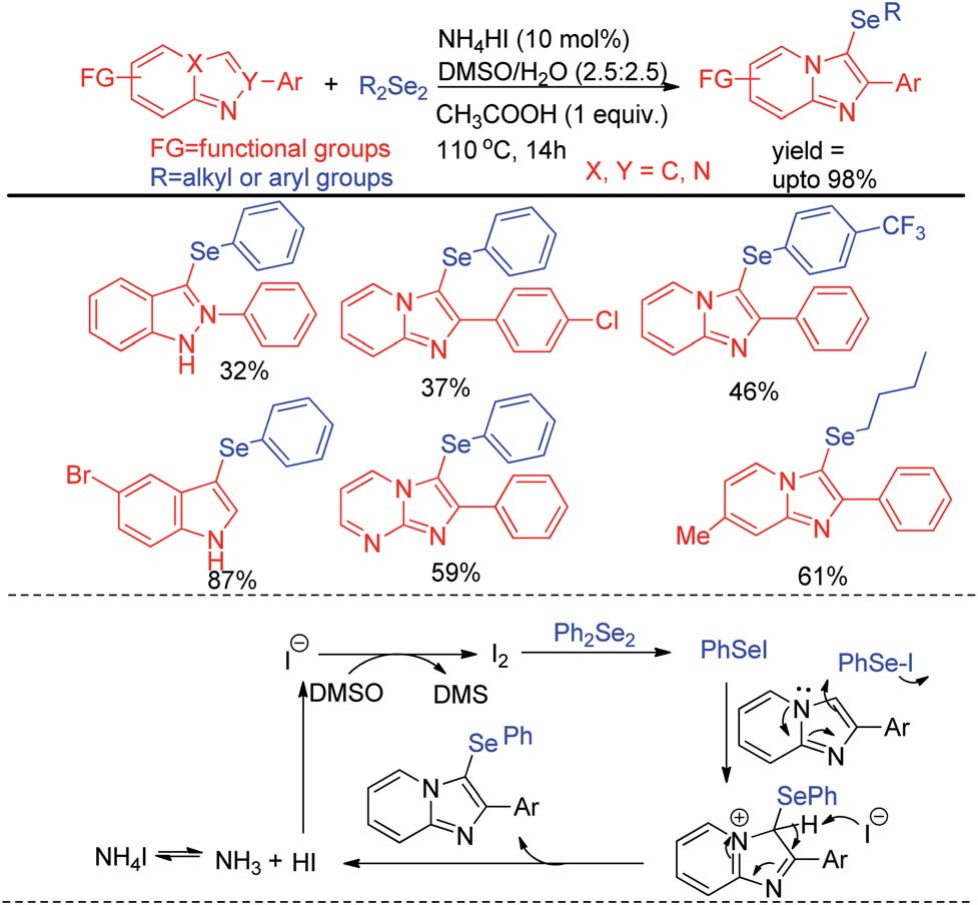
NH_4_I catalysed selenylation of *N*-containing heteroarenes.

Braga *et al.* have reported ammonium iodide (NH_4_I) catalyzed protocol for the synthesis of selenylimidazo[1,2-*a*]pyridines from *N*-heteroaryls and diphenyldiselenide in DMSO using acetic acid as an additive (Scheme 17).^33^ This protocol is highly efficient for the selenation of different 5-membered *N*-heteroaryls *e.g.* indole, imidazothiazole, indazole and imidazopyrimidine having different substituents in the aromatic ring.

**Scheme 18:**
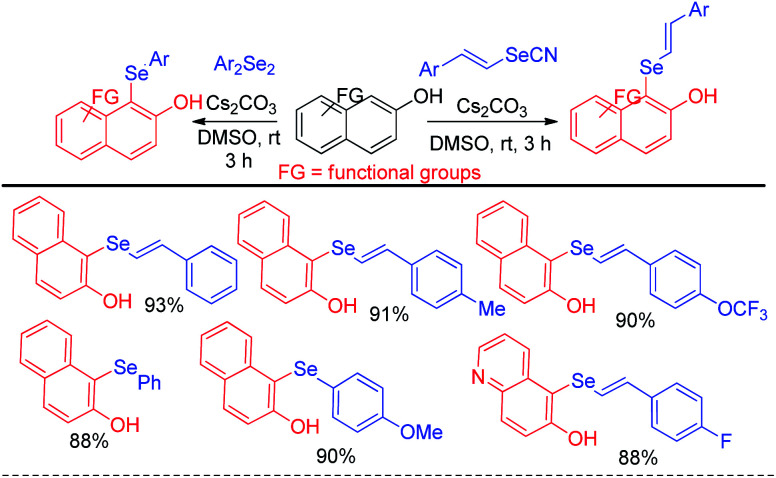
C-1 selenylation in 2-hydroxy naphthalene derivatives under mild conditions.

The reaction proceeded through the *in situ* formation of iodine by oxidation of iodide by DMSO which converted diselenide into active electrophile organo-seleniumiodide. Electrophilic attack by heteroarenes led to the formation of product and the resultant iodide took part in another cycle. Ranu *et al.* have reported an efficient method for the synthesis of bicyclic di-aryl and styrenyl/aryl selenides by using 2-naphthanol and diarylselenides/sterenylselenocyanate respectively without using any oxidant or additive at room temperature (Scheme 18).^34^

**Scheme 19:**
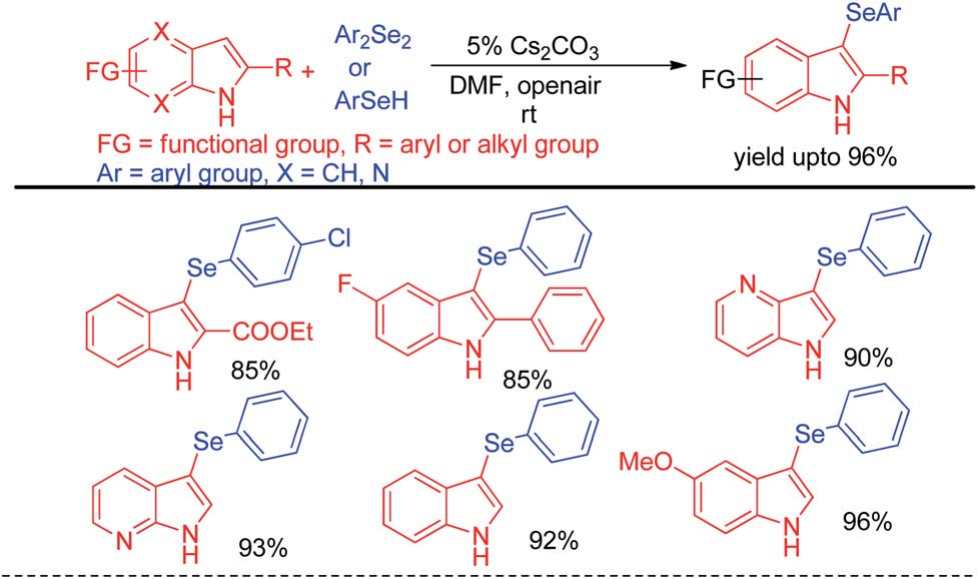
Base mediated selenylation of N-heterocycles under air.

Selenation occurs at the 1-position of 2-naphthols through C–H bond functionalization in dimethyl sulfoxide (DMSO) in presence of Cs_2_CO_3_ as base. A series of 2-naphthanol containing both electron donating and withdrawing groups reacts with both diaryldiselenide and vinyl selenocyante successfully to give corresponding napthtyl-aryl selenides and napthyl vinyl selenides respectively by following an electrophilic aromatic substitution mechanism in good to excellent yields. Jana and co-workers have reported an interesting base promoted selenylation in the electron rich position of indole systems with phenyl selenol under oxygen atmosphere in room temperature without using any external oxidant (Scheme 19).^35^ Aryl selenols with different substituents in the aromatic ring have been employed for the synthesis of library of aryl/heteroaryl selenides through Friedel–Crafts type electrophilic aromatic substitution in electron rich position of heteroarenes such as indole, 1*H*-pyrrolo[3,2-*b*]pyridine, 1*H*-pyrrolo[2,3-*b*]pyridine *etc.* The authors have proposed an ionic mechanism for this reaction which was established with the help of EPR studies.

**Scheme 20:**
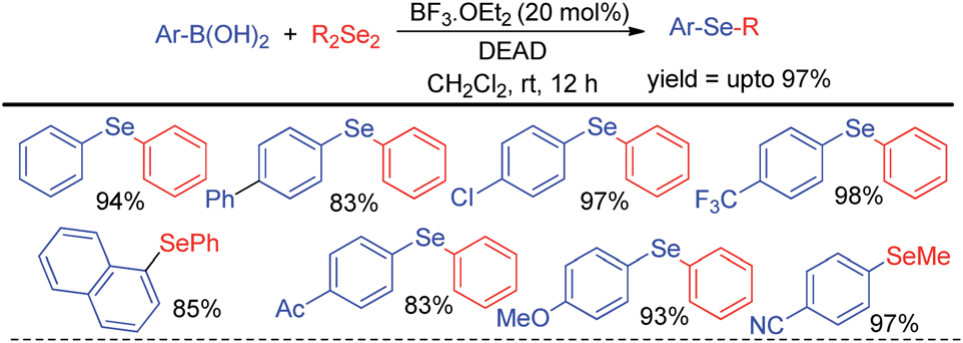
Acid catalyzed reaction of boronic acids with diselenides in presence of DEAD.

Zhao and co-workers reported an acid catalyzed oxidative cleavage of Se–Se bonds of organodiselenides in presence of DEAD and applied this process for the reaction of aryl boronic acids with diselenides leading to the formation of a library of diaryl or aryl alkyl selenides (Scheme 20).^36^ Authors applied BF_3_ as Lewis acid for this reaction. The reaction was also explored with disulfides for the synthesis of organosulfides.

**Scheme 21:**
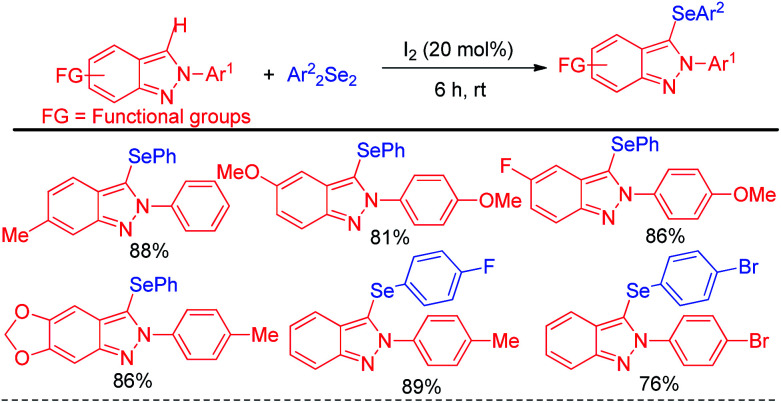
Iodine catalyzed selenylation in 2*H*-indazoles under solvent free conditions.

The concept of iodine catalysed activation of organodiselenides was successfully applied by Hajra *et al.* towards electrophilic selenylation of 2*H*-indazoles for the synthesis of a library of 3-(phenylselanyl)-2*H*-indazoles (Scheme 21).^37^ The reactions were performed under room temperature in absence of any solvent with large functional group compatibility in the aromatic rings of both reactants.

**Scheme 22:**
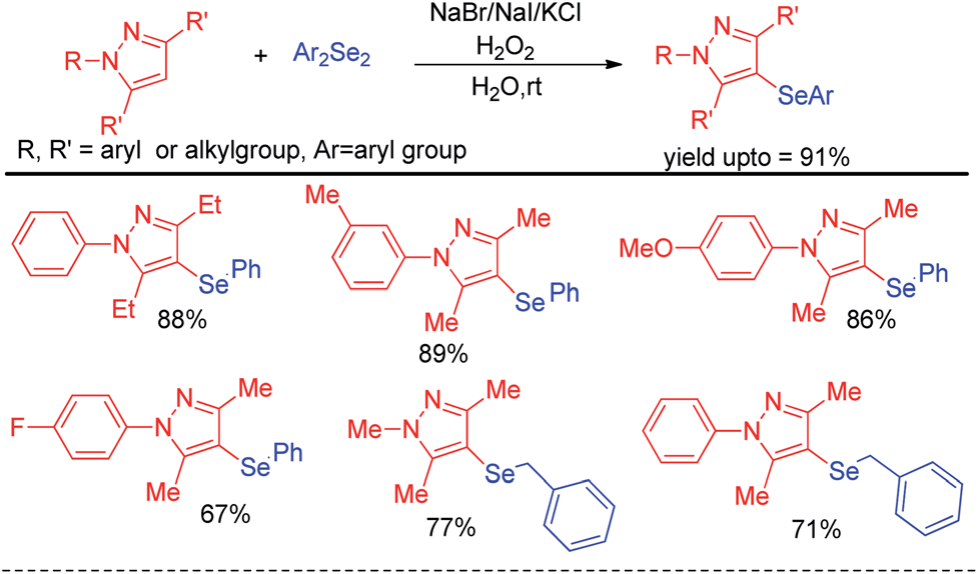
Halide salt promoted synthesis of 4-selenyl pyrazoles.

Yan *et al.* reported haloid salt (bromide and iodide) promoted protocol for the preparation of 4-selanylpyrazoles from *N*-protected pyrazoles in presence of hydrogen peroxide as oxidant in water at room temperature (Scheme 22).^38^

**Scheme 23:**
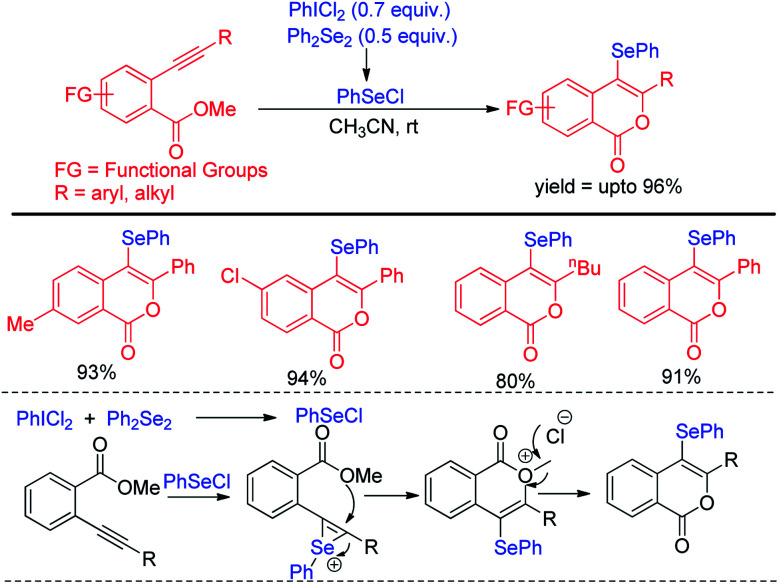
One-pot regioselective synthesis of 4-chalcogenylisocumarins.

The protocol was successful in synthesizing a series of 4-selenyl pyrazoles in moderate to good yields. According to the authors haloid anion reacts with oxidant and forms molecular halogen which helped to break Se–Se bond during the reaction to form active electrophile PhSeX which underwent reaction following electrophilic aromatic substitution in pyrazoles. In 2019, Yunfei and co-workers have introduced synthesis of 4-selenyl isocoumarins from *o*-(1-alkynyl)benzoates and (*Z*)-2-alken-4-ynoates *via in situ* formation of organoselenium halides from organodiselenides and dichlorophenyliodonium salts (Scheme 23).^39^ The one pot strategy resulted regioselective synthesis of a library of 4-selenyl isocoumarins in good to excellent yields under mild reaction conditions with high functional group compatibility.

The *in situ* formed organoselenium halide activated the triple bond in substrate and thus the intramolecular cyclization resulted the formation of product.

**Scheme 24:**
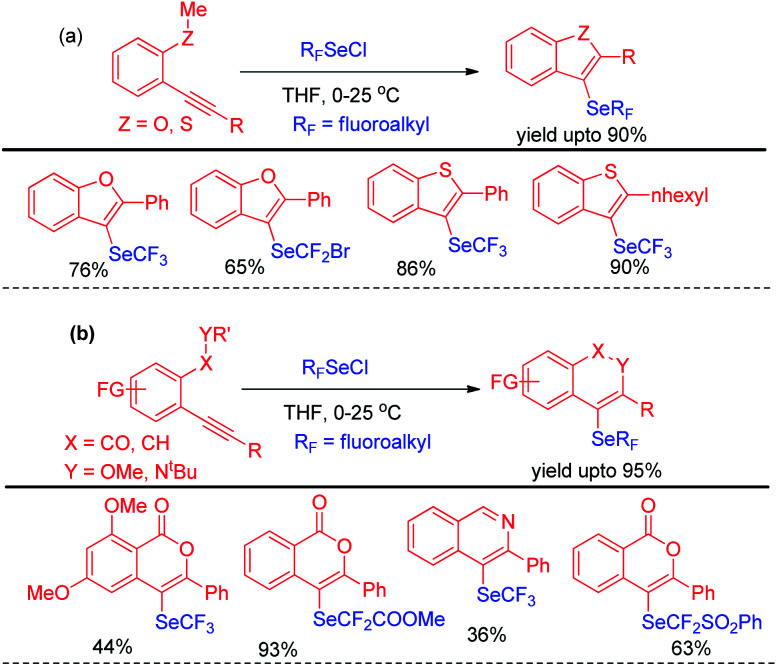
Synthesis of fluoroalkylselenolated heterocycles under room temperature.

**Scheme 25:**
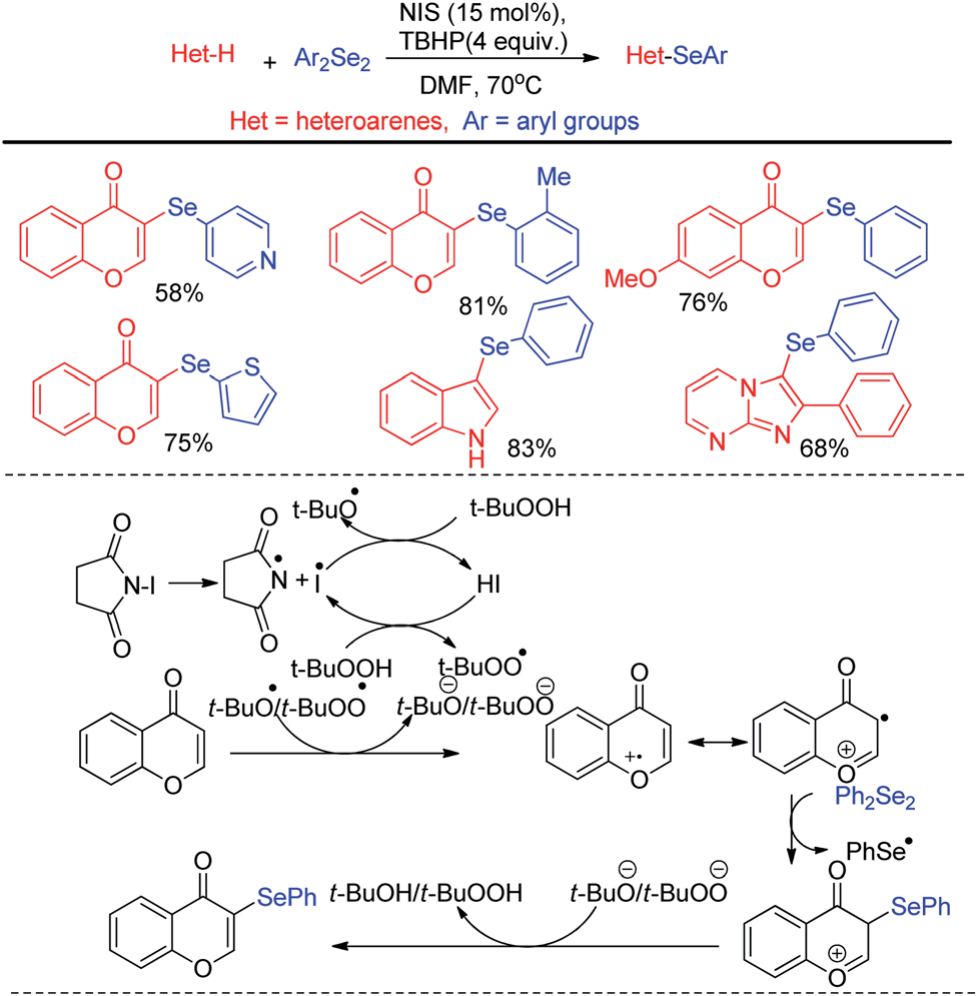
I_2_/TBHP mediated C–H selenation of heteroarenes in room temperature.

Billiard *et al.* developed an interesting strategy for the synthesis of fluoroalkylselenolated heteroarenes and heterocycles by involving *in situ* formed fluoroalkylselenylchloride into tandem cyclization with *ortho*-substituted alkynes (Scheme 24).^40^ Different fluoroalkylselenolated five [Scheme 24a] and six [Scheme 24b] membered heterocycles including benzofurans, benzothiophenes, isoquinolines, isocoumarins *etc.* were synthesized in good to excellent yields by this protocol under room temperature. Zengqiang Song and co-workers have developed a C(sp^2^)–H selenation of different heteroacycles with diselenides (0.5 equiv.) NIS as catalyst and TBHP as additive (Scheme 25).^41^

**Scheme 26:**
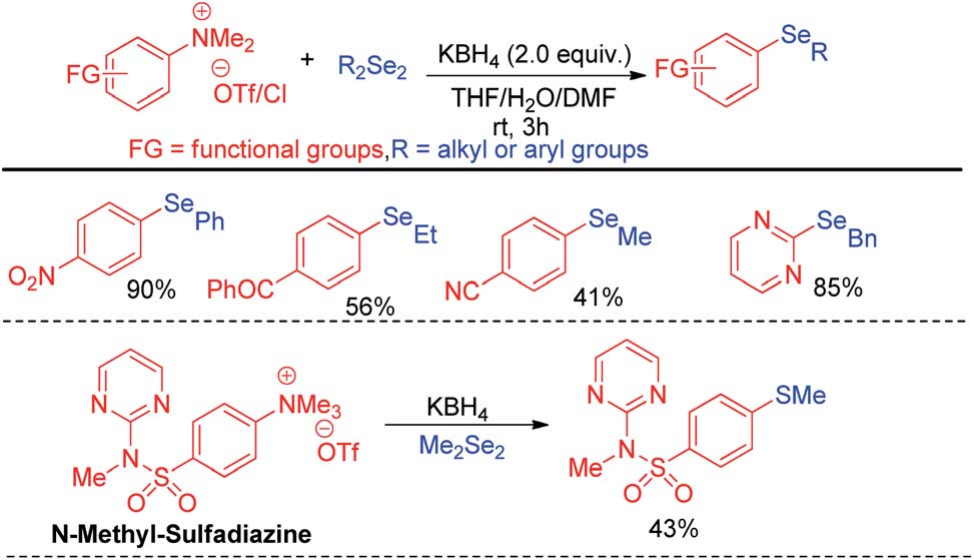
Aryl C–Se bond formation by C–N bond cleavage from aryl ammonium salts.

A wide range of heteroarenes such as flavones, indoles, imidazo[1,2-*a*]pyridines, imidazo[2,1-*b*]thiazoles underwent reaction with diaryl/alkyl/benzyl diselenides to produce good yields of products. A radical mechanistic approach for this reaction was proposed by the authors where *tert*-butylperoxyl or *tert*-butoxyl radicals are generated by NIS and take part in SET process from heteroarenes to initiate the reaction. Zhang and co-workers very recently reported an innovative idea of aryl C–heteroatom bond formations *via* C–N bond cleavage using aryl ammonium salts as active electrophile (Scheme 26).^42^ The authors applied the idea in aryl C–Se bond formations by using organodiselenides in presence of KBH_4_ as base under room temperature. The authors proposed S_N_Ar mechanism for the reaction. Although the reaction required activating electron withdrawing substituents in aromatic ring to facile the reaction. The authors also explored the scope of the reaction by synthesizing selenium derivative of antibiotic drug sulfadiazine.

**Scheme 27:**
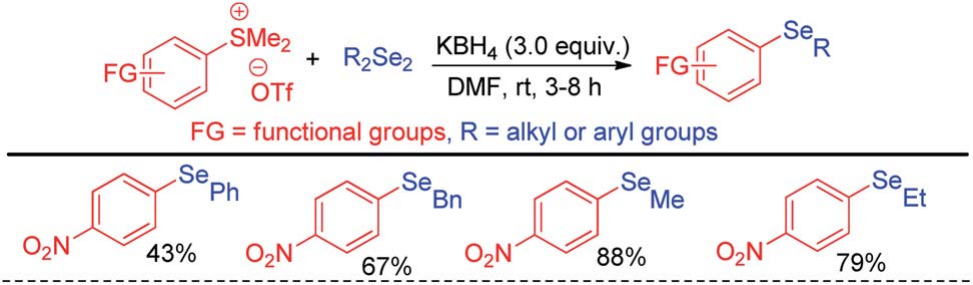
Aryl C–Se bond formation by C–S bond cleavage from aryl sulfonium salts.

The same group later explored the aryl carbon–heteroatom bond formations by C–S bond cleavage by using aryl sulfonium salts as electrophiles by S_N_Ar mechanism.^43^ Thus the authors have explored aryl C–Se bond formation by C–S bond cleavage using organodiselenides in presence KBH_4_ as base like previous protocol under room temperature (Scheme 27).^43^ The authors only explored the scope of the reaction with *para*-NO_2_-phenyl sulfonium salts. Thus although the reaction condition is mild the protocol suffered from limited substrate scopes due to the requirement of presence of specific activating groups in aryl ring.

**Scheme 28:**
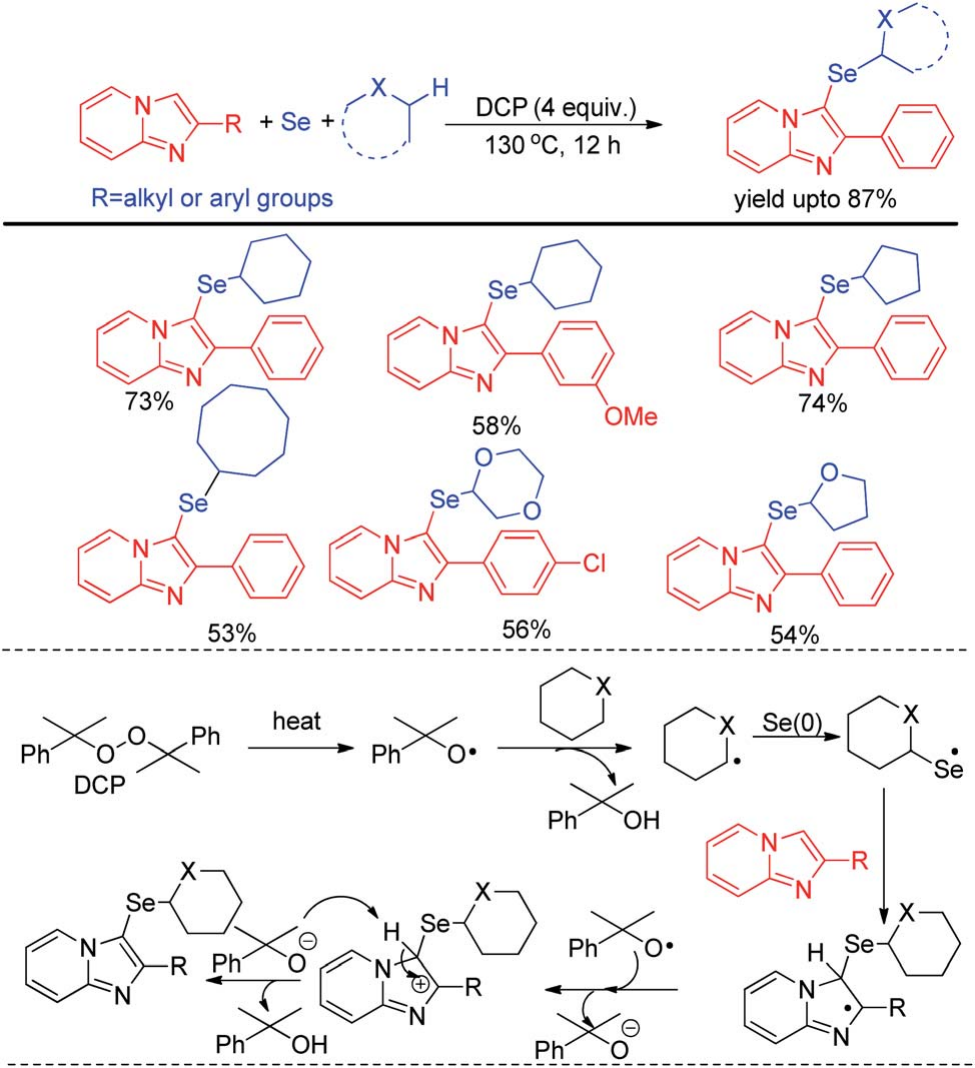
DCP mediated C–H selenation of imidazopyridines under room temperature.

Zhao and co-workers recently reported C–H selenation of cyclic alkanes and ethers by using selenium powder in presence of 4 equivalent DCP as oxidant (Scheme 28).^44^ The reaction followed a radical mechanism where DCP acted as radical initiator. The reaction was found to form trace amount of product in presence of TEMPO. Cyclic alkanes such as cyclopentane, cyclohexane, cycloheptane, cyclooctane was found to produce moderate to good yields of 3-selenoalkyl imidazopyridines under the reaction conditions. Cyclic ethers such as 1,4-dioxane, THF *etc.* were also found to react successfully under the reaction conditions.

**Scheme 29:**
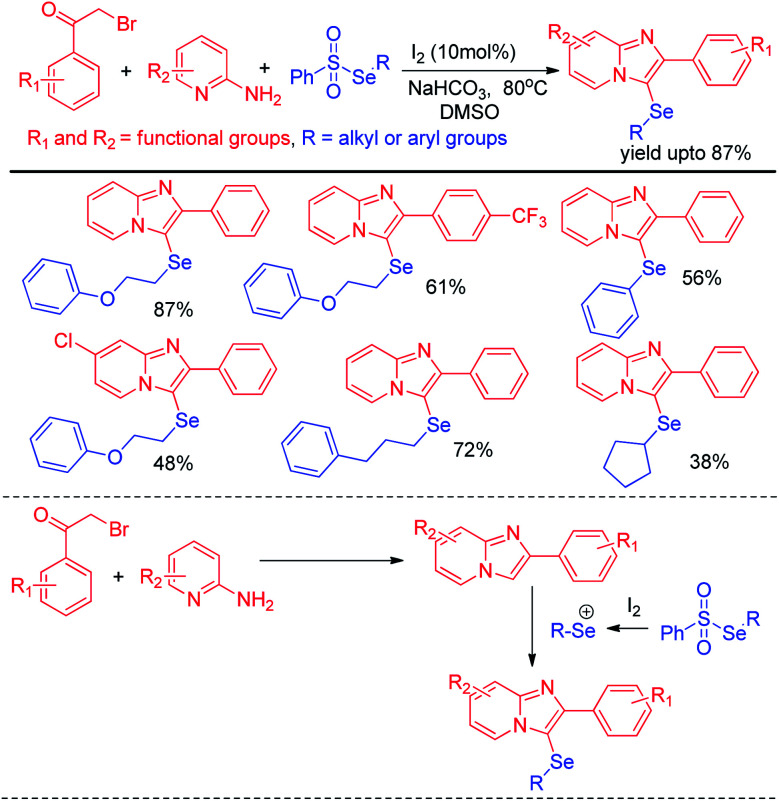
Iodine catalysed one pot synthesis of 3-selenylimidazo[1,2-*a*]pyridine.

**Scheme 30:**
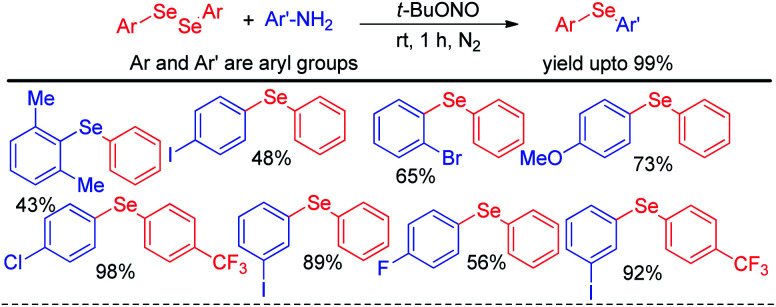
Synthesis of diaryl selenides from aromatic amines under solvent free conditions.

Selenosulfonates were introduced as effective organoselenium source by Wang and Ji *et al.* for the synthesis of 3-selenylimidazo[1,2-*a*]pyridine derivatives *via* iodine promoted one pot multicomponent reaction with pyridine-2-amines and aryl methyl ketones (Scheme 29).^45^ Selenosulfonate in presence of iodine produced organoselenium cation intermediate which underwent electrophilic attack by *in situ* formed imidazo[1,2-*a*]pyridine to form the product. Very recently Lee and co-workers have reported a reaction of aromatic amines with diaryldiselenides in presence of ^*t*^BuONO as diazotizing agent under neat conditions in room temperature (Scheme 30).^46^ The authors were able to synthesize a number of diaryl selenides with different substituents in aromatic ring. However blue light irradiation was required in case of sulphides.

**Scheme 31:**
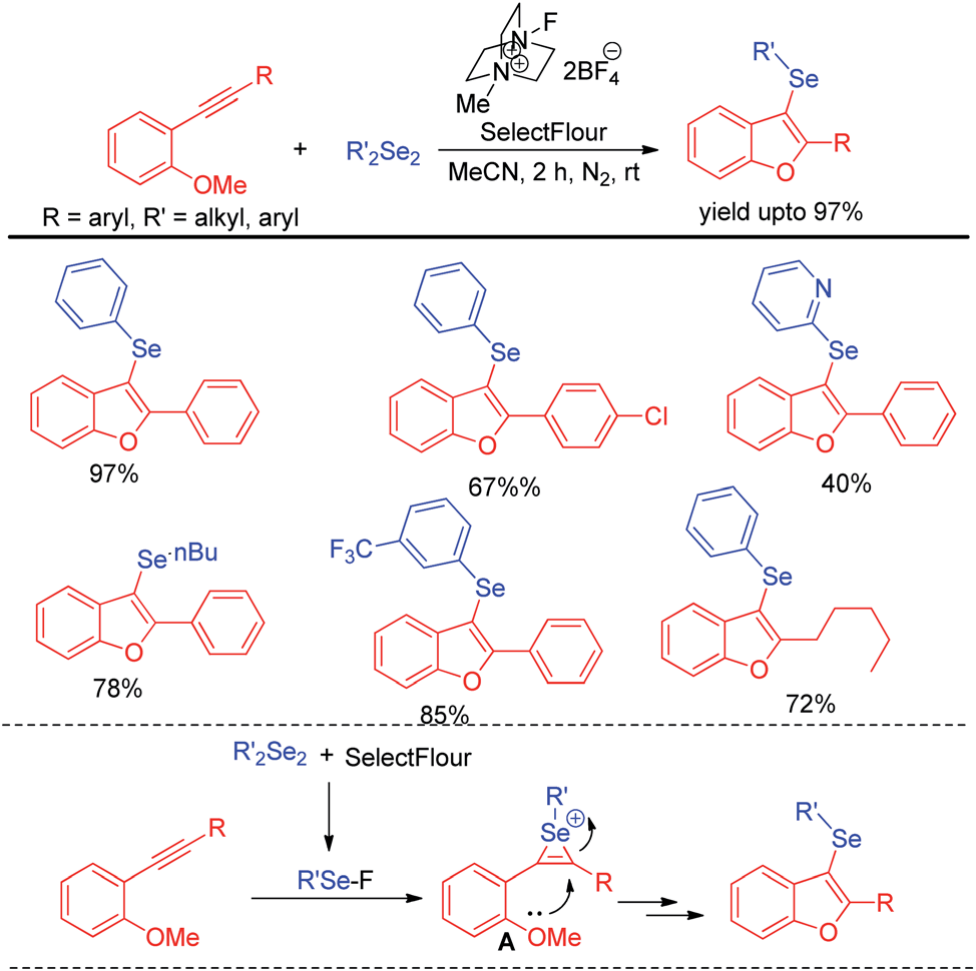
Selectfluor promoted synthesis of 3-selanylbenzo[*b*]furans.

Very recently Schumacher & Silva *et al.* have revealed the role of selectfluor in generating electrophilic selenium species from organodiselenides and thus explored its application for the synthesis of 3-selanylbenzo[*b*]furans *via* electrophilic cyclization of 2-organoalkynyl anisoles under nitrogen atmosphere (Scheme 31).^47^ diaryl, di-heteroaryl, dialkyl diselenides were compatible under the reaction conditions to produce moderate to excellent yields of products. Organoselenides in presence of selectfluor produced organoselenium fluorides as active electrophile which underwent electrophilic attack by triple bond to form intermediate A. Finally desired product was obtained by intramolecular cyclization from A. The authors also explored the scope of this reaction in synthesizing 3-selenylindoles from 2-organoalkynyl anilines.

**Scheme 32:**
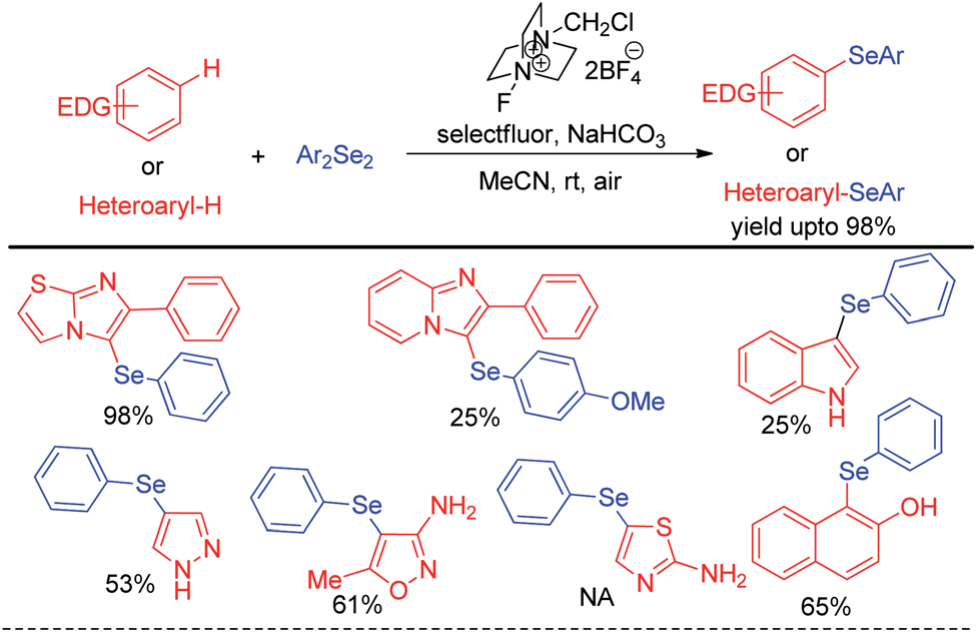
Selectfluor activated C–H selenation of heteroarenes and electron-rich arenes.

The concept of activation of organodiselenides by selectfluor was further applied by the same author towards the C–H selenation of heteroarenes and electron rich aromatic compounds (Scheme 32).^48^

The reactions were performed under air in room temperature in acetonitrile solvent. A large array of heteroarenes such as, imidazo[2,1-*b*]thiazole, imidazo[2,1-*b*]pyridine, indole, pyrazole, thiazole *etc.* underwent selenation at their electron rich positions successfully by following an electrophilic substitution mechanism. However oxazole was found to be inactive towards electrophilic selenation under this protocol.

## 3 Under photoinduction or visible light medium

**Scheme 33:**
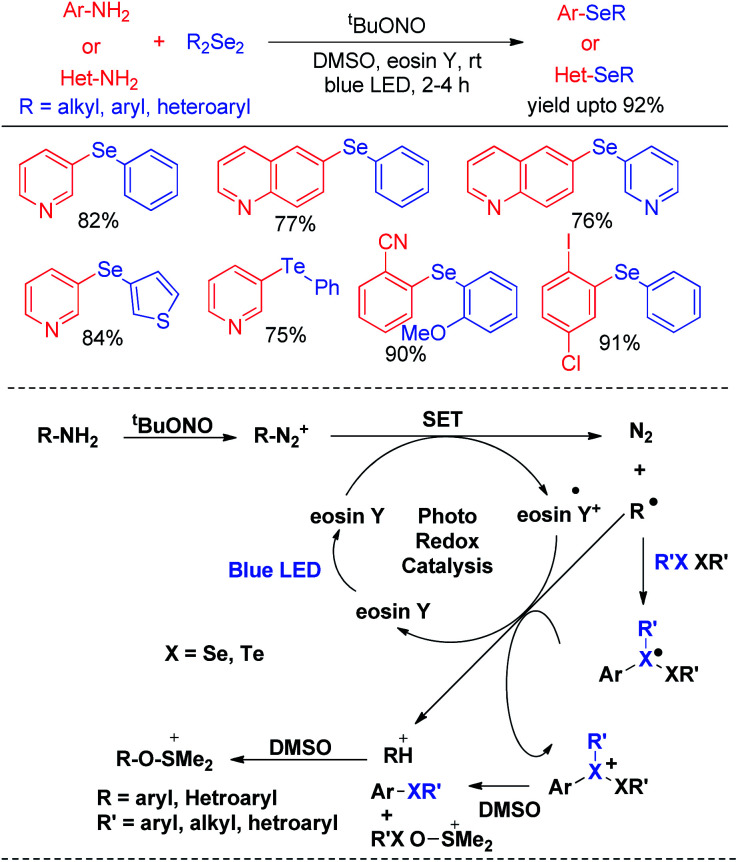
Visible light photocatalyzed synthesis of diatyl/heteroaryl selenides/tellurides from aromatic amines.

In last decade visible light has appeared as a sustainable and economic power source which initiates a large array of organic reactions based on C–C and C–heteroatom bond formations in presence of transition metal complexes or organic dyes as photocatalysts under room temperature. Such protocols are highly green as the energy source is non-toxic, highly abundant and without any waste generating unlike burning of fossil fuels. Hence the concept of visible light photocatalysis was applied for the synthesis of aryl and heteroaryl selenides and tellurides by C–Se/C–Te bond forming reactions in recent years. In 2014, Ranu and co-workers have developed one pot synthesis of unsymmetrical diaryl/heteroaryl selenides from aryl/heteroaryl amines by *in situ* diazotation with and *tert*-butyl nitrite under blue LED light using eosin Y as photocatalyst at room temperature (Scheme 33).^49^ The reactions are high yielding which makes the procedure suitable for synthesis of a wide range of aryl/heteroaryl (*e.g.* pyridine, thiophene, quinoline, thiazole *etc.*) selenides. They also explored the scope of this methodology for the synthesis of diaryl tellurides and sulphides. Differential bi-selenation in the same aromatic ring was also performed by starting with nitroanilines. The authors have proposed the DMSO driven radical mechanistic approach based on single electron transfer from photocatalyst under blue light for this reaction. The authors found that the reaction was quenched in presence of TEMPO under reaction conditions.

**Scheme 34:**
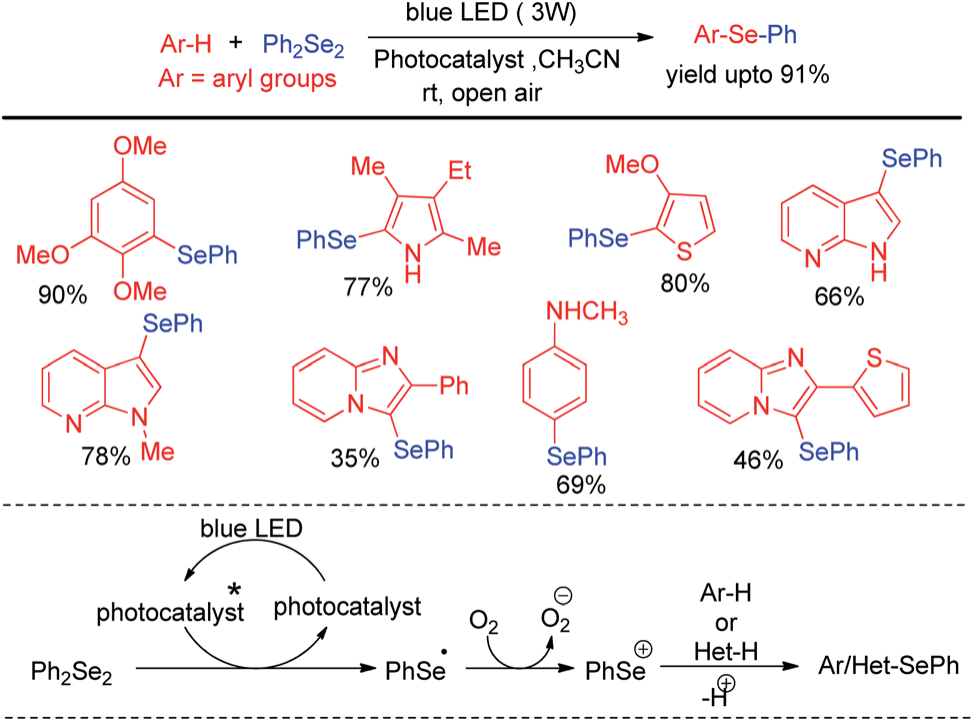
Visible light mediated C–H selenation of electron rich arenes/heteroarenes under air.

Liu and co-workers reported C–H selenation of electron rich arenes and heteroarenes with diselenides under aerobic oxidation in room temperature (Scheme 34).^50^ The authors used Ir-complex (bis[2-(4,6-difluorophenyl)pyridinato-*C*2,*N*](picolinato)iridium(iii)) (Flrpic) as photocatalyst for this reaction. However the authors also established that the reaction was also successful with organocatalyst Na_2_-eosin-Y which was found less efficient by a marginal scale under the reaction conditions.

**Scheme 35:**
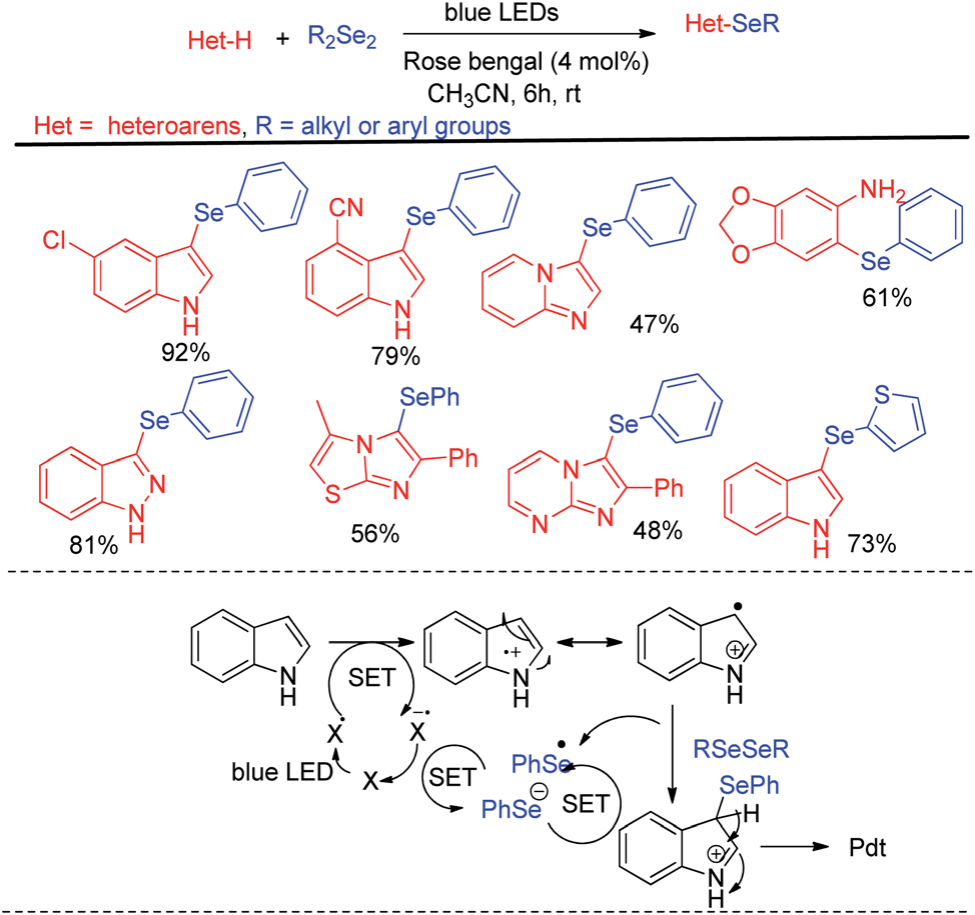
Rose Bengal catalysed C–H selenation of heteroarenes under room temperature.

Different electron rich arenes and heteroarenes such as thiophene, pyrrole, indoles, imidazopyridine, methoxybenzenes were found to undergo C–H selenation to produce good to excellent yields. Less electron rich arenes such as azulene was also found to undergo selenation under the reaction conditions with lower yield production. The authors established the mechanism on the basis of nucleophilic attack by arenes on PhSe+ which was generated from diselenide by single electron transfer from photocatalyst under blue-LED followed by oxidation by oxygen. Braga *et al.* have developed visible light photocatalyzed synthesis of selenyl indole, selenylimidazoles and selenylarenes *via* direct C(sp^2^)–H functionalisation by using Rose Bengal as photocatalyst (Scheme 35).^51^ This is an atom economic & regioselective reaction catalysed by Rose Bengal under blue LEDs using CH_3_CN solvent at room temperature under both argon and oxygen atmosphere. Different kinds of heterocycles such as indoles, benzopyrazines, imidazo[1,2-*a*]pyridines, imidazo[1,2-*a*]pyrimidines, imidazo[2,1-*b*]thiazoles and electron rich arenes underwent reaction with diselenides to produce good to excellent yields following a photocatalyzed radical mechanistic pathway. The authors failed to initiate any conversion to the products in case of 3-substituted indoles. However several *N*-protected indoles were found to generate selenated products in good yields. The reaction failed to initiate any conversion to the product with ditellurides and disulfides.

**Scheme 36:**
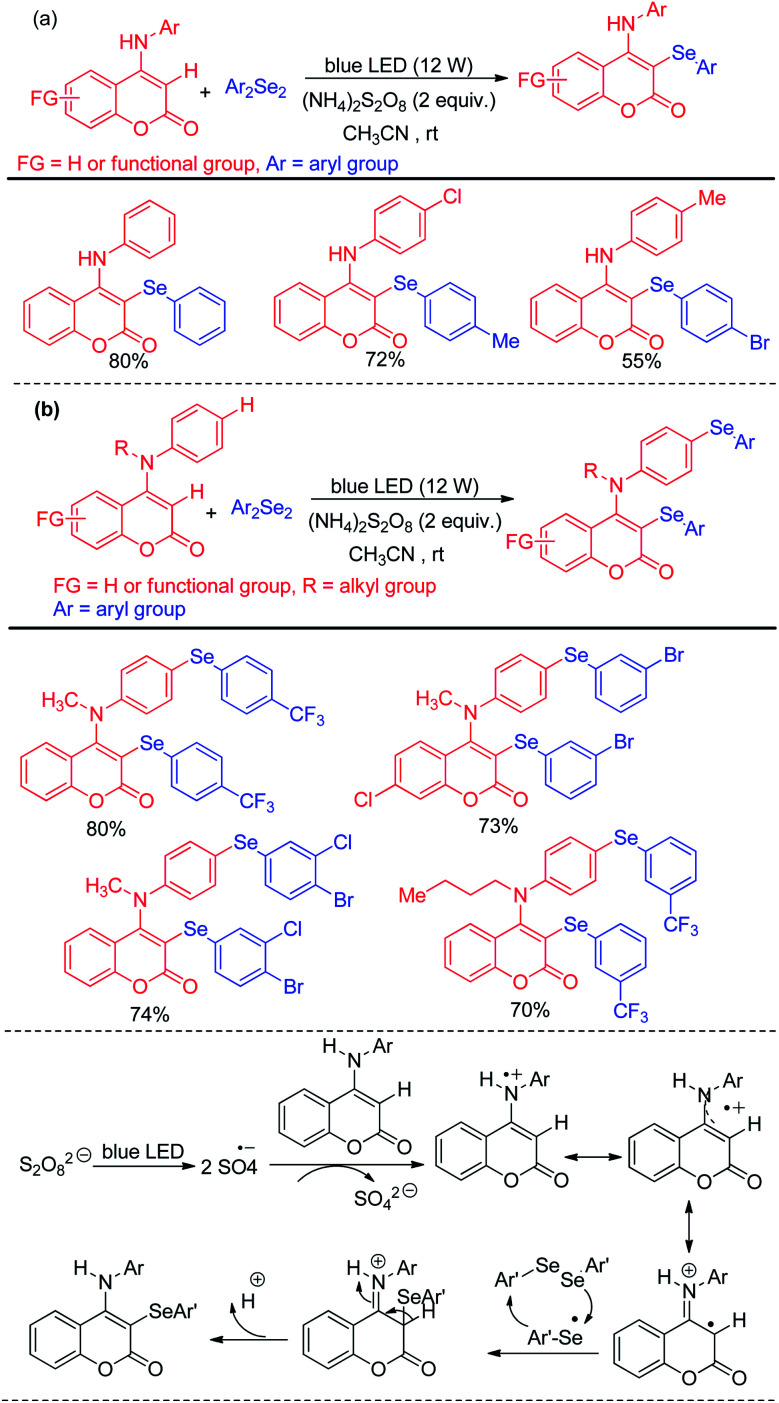
(NH_4_)_2_S_2_O_8_ driven selenylation of 4-amino coumarins under blue light.

Yang *et al.* have reported visible light mediated regioselective selenylation of 4-amino substituted coumarin derivatives by diselenides without using any photocatalyst. CH_3_CN was used as a solvent under aerial condition under blue LED light (Scheme 36a).^52^ (NH_4_)_2_S_2_O_8_ was used as an oxidant. Although *N*-aryl amino substituted coumarins underwent selenylation successfully, the authors failed to achieve any conversion to the product with –NH_2_ and –NHMe substituted coumarins under the reaction conditions. The authors also achieved dual selenylation selectively by reacting *N*-substituted 4-(phenylamino)-2*H*-chromen-2-one derivatives as substrates (Scheme 36b). The reaction showed excellent functional group tolerance for this reaction. The oxidant (NH_4_)_2_S_2_O_8_ formed sulfate radical anion intermediate under light which took part in single electron transfer process to initiate the reaction.

**Scheme 37:**
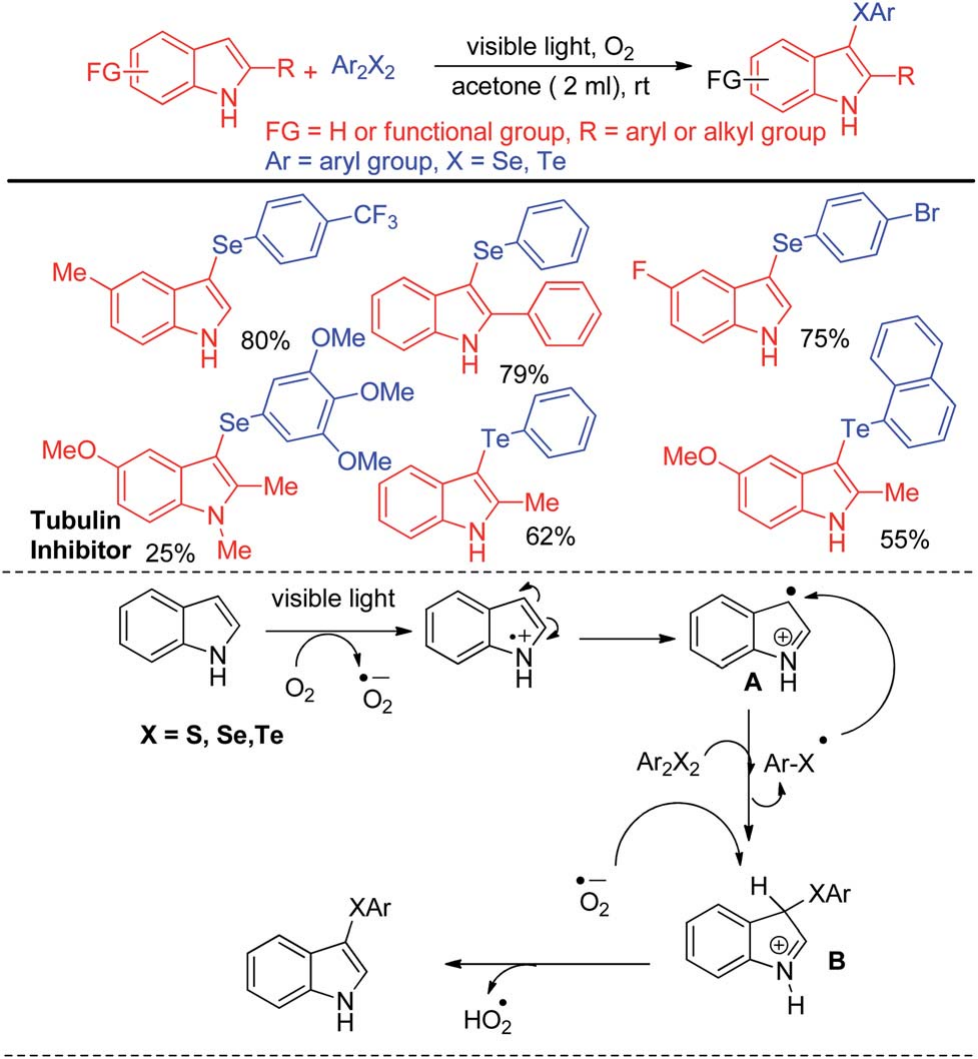
Visible light mediated catalyst free 3-organochalcogenation of indoles.

Recently Kumar & co-workers have reported visible light mediated organoselenylation of indoles by diaryl selenides in acetone solvent under base and catalyst free conditions at room temperature in presence of oxygen (Scheme 37).^53^ The authors have used 26 W CFL bulb as light source and acetone as solvent. Several substituted indoles and electron rich arenes underwent selenylation by this protocol by producing 3-organoselenyl indoles in excellent yields under mild conditions. The authors have successfully applied this protocol for performing reaction with diarylditellurides towards the synthesis 3-tellenylindoles which was achieved for the first time under light. However the yields were lower in comparison to selenylation process. The authors also explored the scope of this reaction with diaryldisulfides and ammoniumthiocyanate to produce 3-sulfenyl indoles and 3-thiocyanoindoles respectively. The protocol was successfully applied for the synthesis of tubulin inhibitor. The reaction proceeded through the formation of radical cation intermediate A from indole by SET from indole to oxygen under visible light. As dichalcogenides are prone to radical attack, usual radical attack from A to dichalcogenides led to the formation of B which was converted into product *via* water exclusion by oxygen radical anion.

**Scheme 38:**
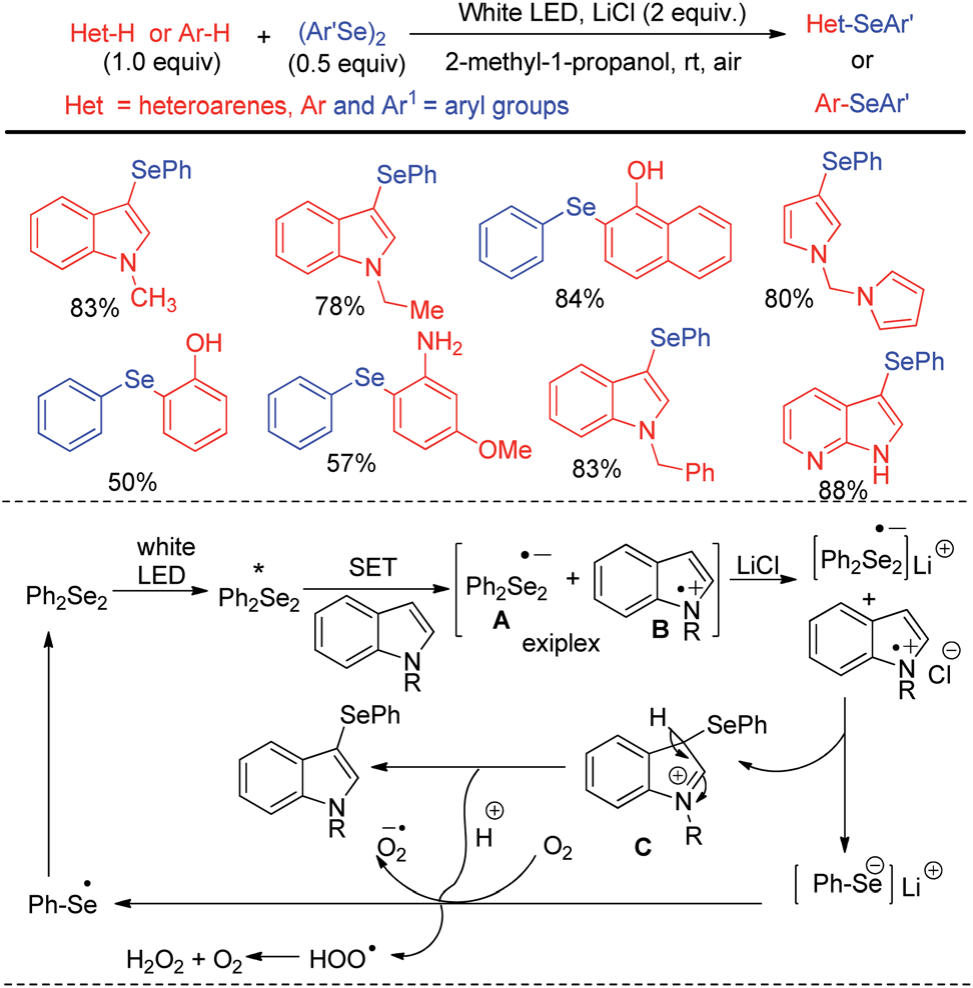
LiCl promoted C–H selenylation of electron rich arenes and heteroarenes under white LED.

**Scheme 39:**
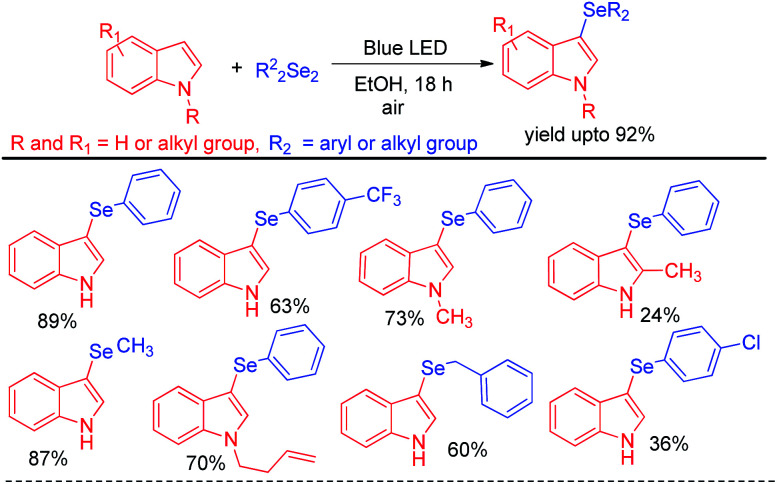
Visible light mediated catalyst free C–H selenation under air.

Kumaraswamy *et al.* proposed an atom economical LiCl promoted direct regioselective C(sp^2^)–H selenation of indoles, imidazopyridines and electron-rich arenes under white LED irradiation without using any catalyst or sensitizer at room temperature under open air (Scheme 38).^54^ Using 0.5 equivalent of diselenides w.r.t. arenes or heteroarenes the authors have successfully performed selenylation in several *N*-protected or NH-indoles, pyrroles and electron rich arenes in high yields. However the reaction did not produce any satisfactory result with taking with electron withdrawing *N*-protecting groups such as –boc, –tosyl *etc.* in indoles. The authors proposed radical mechanistic pathway for the reaction where diselenides were getting exited under white LED and electron transfer from indole leads to the formation of an exiplex bearing a radical anion (A) and a radical cation (B) intermediate. LiCl acted as stabilizer of the radical ion pair which indeed generated intermediate C that led to the formation of product by proton transfer towards oxygen radical anion. Heredia and Arguello *et al.* have developed visible light promoted regioselective synthesis of 3-selenylindoles from organic diselenides and indole at room temperature in presence of air without under blue LED in absence of any photocatalyst (Scheme 39).^55^ The reactions were performed in green ethanol solvent. Several diaryl selenides have been also synthesized from electron-rich arenes under this reaction conditions in good to excellent yields. The reaction went through the common light mediated radical mechanistic path where oxygen played a crucial role by taking part in SET process through the formation of peroxo radical.

**Scheme 40:**
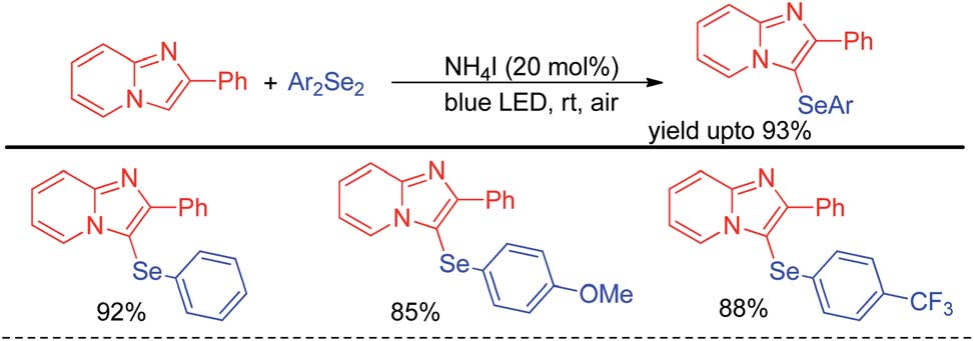
NH_4_I catalysed selenylation of 2-phenyl imidazopyridines under blue LED.

Yasuike *et al.* have reported NH_4_I catalyzed selenylation of 2-phenyl imidazopyridines under blue LED at room temperature under aerial conditions (Scheme 40).^56^ The protocol was successfully applied for the synthesis of various 2-phenyl-3-(arylselanyl)imidazo[1,2-α]pyridines in good to excellent yields.

**Scheme 41:**
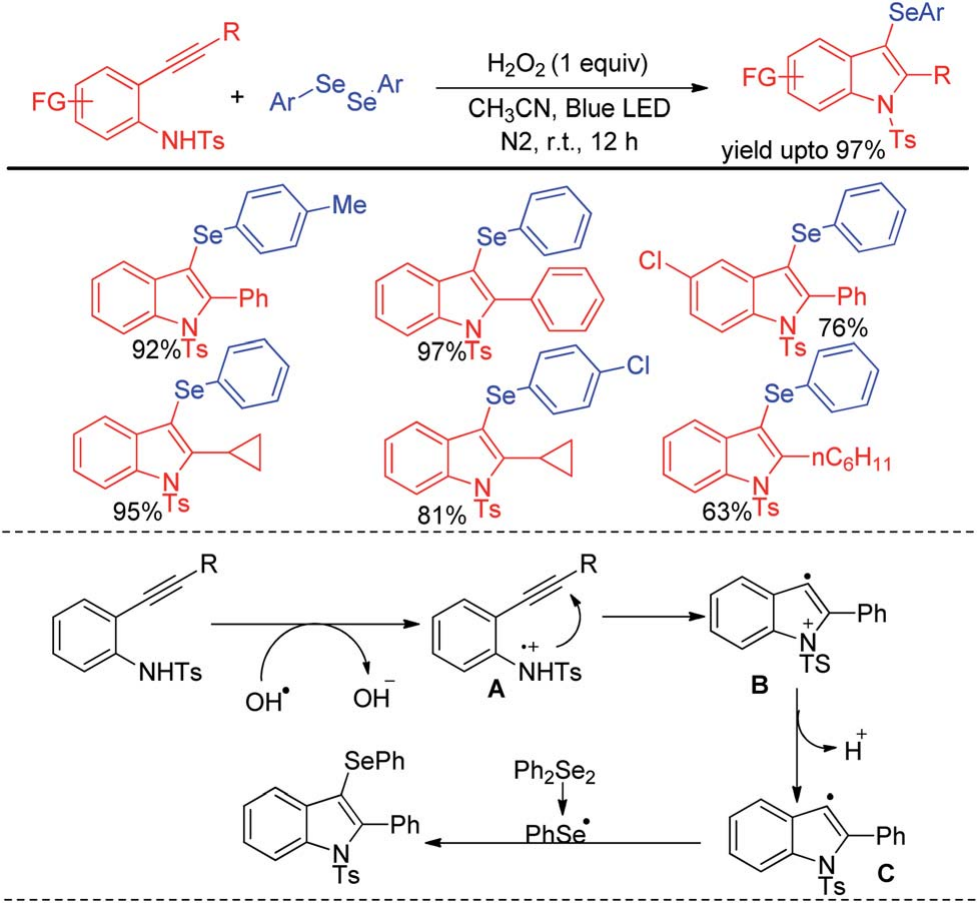
Visible light mediated tandem cyclization of 2-alkynyl anilines in presence of H_2_O_2_.

Wang and coworkers have introduced visible light mediated photocatalyst free oxidative tandem cyclization of 2-alkynyl anilines with diselenides in presence of hydrogen peroxide for the synthesis of 3-selenoindole derivatives (Scheme 41).^57^ The reaction was initiated by hydroxyl radical, generated from hemolytic cleavage of H_2_O_2_ under blue LED. Single electron transfer from 2-alkynyl aniline generates intermediate A which *via* tandem cyclization formed intermediate C radical at the expense of proton. Reaction of C with diselenides produced the desired 3-selenoindoles. The reaction was however reported to be unsuccessful with ditellurides.

**Scheme 42:**
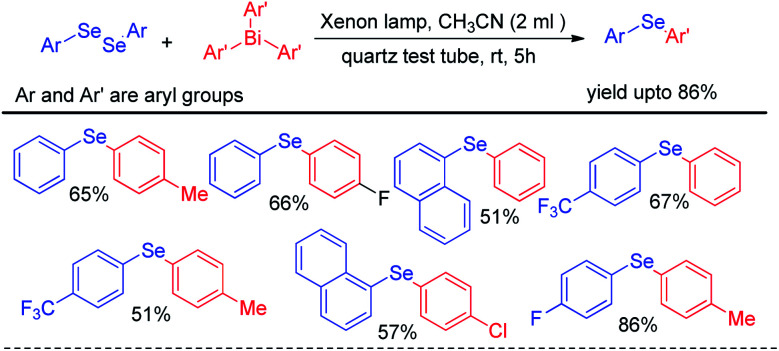
Reaction of triaryl bismuthate with diselenides under xenon lamp irradiation.

Apart from visible light mediated protocols Ogawa and co-workers in 2013 introduced a photoinduced reaction of organodiselenides with triaryl bismuthates for the formation of library of diaryl selenides under xenon lamp irradiation (Scheme 42).^58^ The reactions were performed in quartz test tube under room temperature.

## 4 Under microwave irradiation

**Scheme 43:**
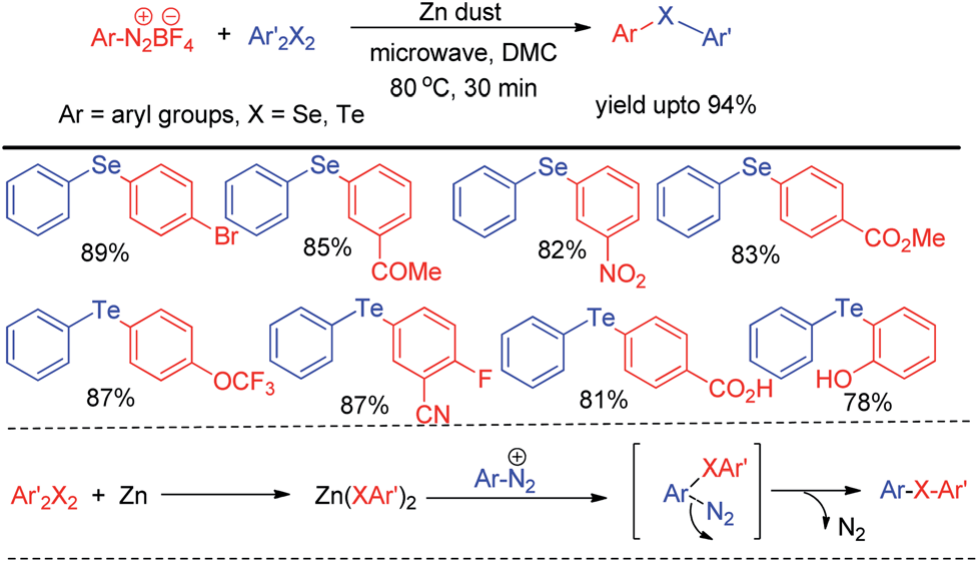
Microwave assisted reaction of aryl diazonium fluoroborate with diselenides.

Microwave assisted organic synthesis has emerged a powerful alternative of reactions under conventional heating due to its several advantages such as uniform heating, rapid reaction speed, absence of wanted side reaction, avoiding environmental heat loss and reduction of producing waste materials. Thus several microwave assisted protocols were developed for the synthesis of organochalcogenides. However the scope of transition metal catalyst free protocols for the synthesis of selenides and tellurides under microwave irradiation is still limited. Ranu *et al.* have reported the synthesis of unsymmetrical diaryl selenides and tellurides by reacting aryl diazonium fluoroborates with organodiselenides and tellurides respectively under microwave irradiation in short reaction time (Scheme 43).^59^

**Scheme 44:**
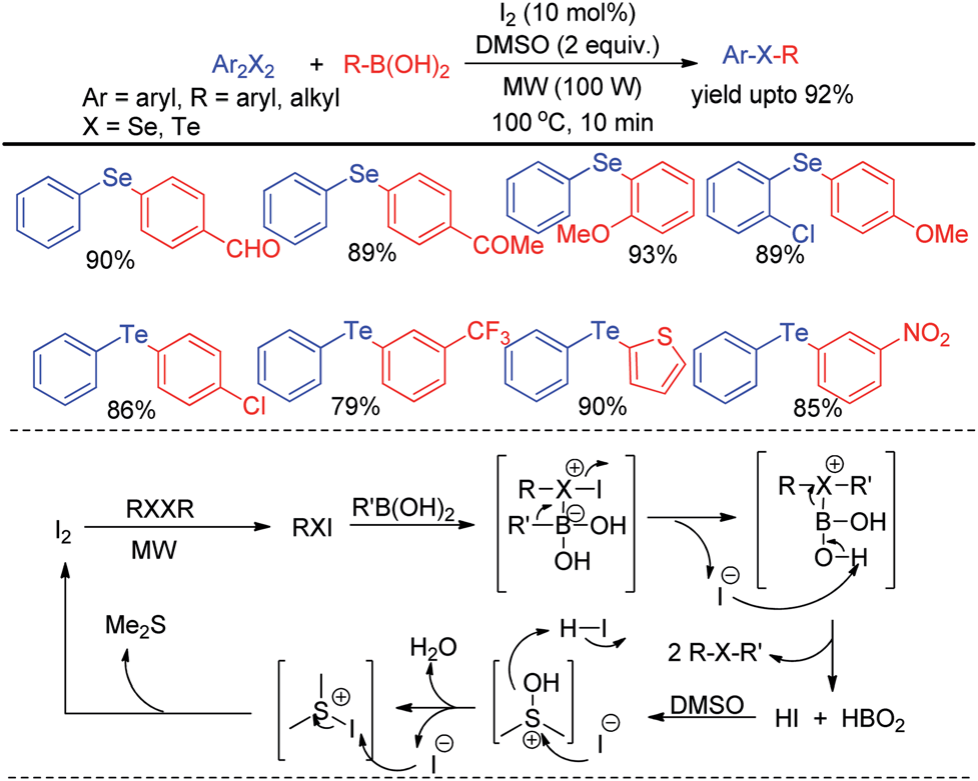
Microwave assisted reaction of boronic acids with diselenides.

The zinc dust was used for the cleavage of diaryldichalcogenides which produced active nucleophile. The striking success of this protocol was its applicability of synthesizing large array of diaryl tellurides which was very less explored previously even by transition metal catalyzed protocols. A wide range of aryl diazonium fluoroborates having both electron donating (–OMe, –OH, –OCF_3_*etc.*) and electron withdrawing groups (–COMe, –COOMe, –CN, –F, –Br, –Cl, –COOH *etc.*) were allowed to react with diphenyl diselenides/tellurides to provide the corresponding diaryl chalcogenides in high yields and high purity within a short reaction time by this protocol. The authors also explored the microwave assisted process for the synthesis of diaryl sulfides. The mechanism was proposed based on nucleophilic attack by Zn(SePh)_2_ on aryl diazonium fluoroborate and the product was obtained at the expense of N_2_. Braga and co-workers have proposed a novel idea of iodine catalyzed synthesis of unsymmetrical diarylselenides/tellurides from boronic acids under microwave irradiation (100 W) in absence of solvent. The coupling occurred in presence of 2 equiv. DMSO as oxidant (Scheme 44).^60^ A library of unsymmetrical diaryl/aryl–heteroaryl selenides and tellurides were synthesized in good to excellent yields with high functional group compatibility. First, diselenide reacts with Iodine to give RSeI as electrophilic intermediate. The possibility of involving radical mechanism was excluded by showing that TEMPO was ineffective in quenching the reaction. Thus the authors proposed an ionic mechanism where the desired product was obtained by nucleophilic attack from chalcogen center to the boron of boronic acid at the expense of HI which by the help of oxidant regenerated iodine.

## 5 Under ball milling

**Scheme 45:**
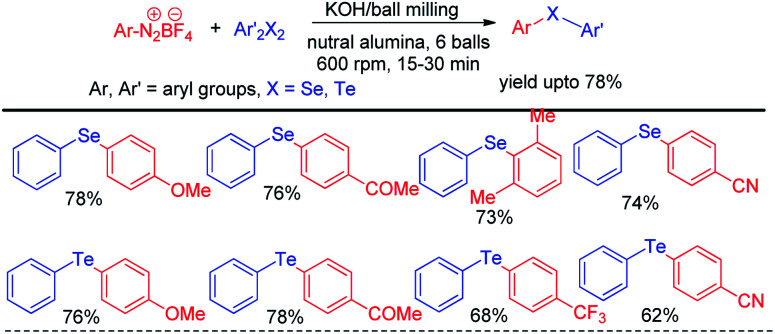
Reaction of aryl diazonium fluoroborates with dichalcogenides under ball milling.

In recent years reactions under ball milling (intense mechanical grinding) have found considerable interest of synthetic chemists due to its greener applications in organic synthesis.^61^ Performing reactions under solvent free conditions, room temperature and no general waste production made it a sustainable tool in synthetic chemistry. Thus ball milling was widely applied in different C–C^62^ and C–heteroatom^63^ bond forming reactions in recent times for the synthesis of various important organic molecules for greener advancement w.r.t. their previously reported protocols. Ranu and coworkers have reported solvent free reaction of aryl diazonium fluoroborates with diaryldiselenides/tellurides for the synthesis of corresponding diaryl chalcogenides under ball milling in room temperature by using KOH as base (Scheme 45).^64^ The main success of this protocol was it did not require formal work up or column chromatography. The product was obtained by simple elution of reaction mixture by ethanol or ethyl acetate followed by evaporation of solvent. Aryl diazonium fluoroborates with different electron donating and withdrawing groups in the aromatic ring were successfully converted into products in good moderate to good yields. The authors performed ball milling operation in inverted rotation directions, with an interval of 10 min and an interval break of 30 seconds. The authors also explored the scope of the reaction for the synthesis of diarylsulphides and dithiocarbamates. The authors have used neutral alumina and solid phase reaction medium under ball milling chamber.

**Scheme 46:**
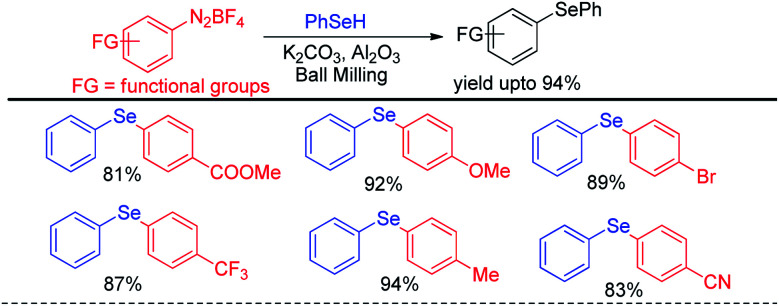
Ball mill assisted reaction of phenyl selenols with diazonium salts under base free conditions.

The same group later explored the reaction with phenyl selenols with aryl diazonium fluoroborates under ball milling where they were able to find better yields for the synthesis of diaryl selenides in presence of milder base K_2_CO_3_ (Scheme 46).^65^

## 6 Under ultrasound

**Scheme 47:**
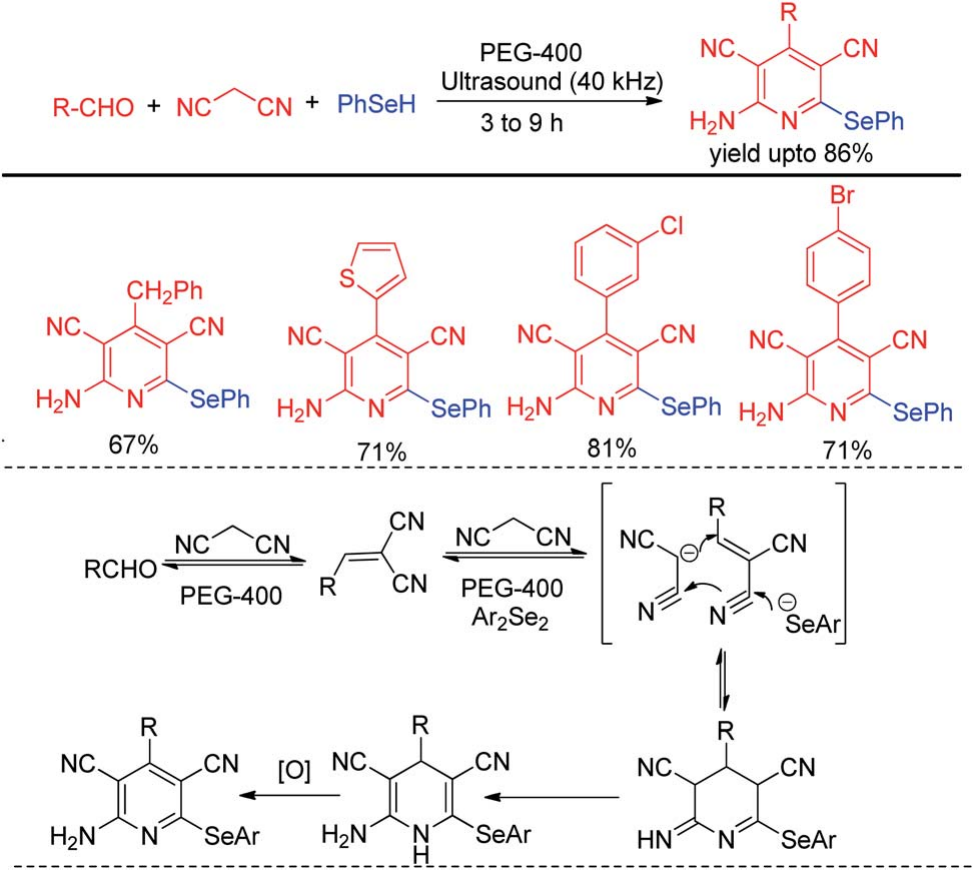
One pot synthesis of 2-aminoselenopyridines *via* ultrasound assisted MCR.

Choudhury and co-workers have reported a simple and sustainable protocol for one-pot synthesis of 2-amino selenopyridines by performing multicomponent reaction of aldehydes, malononitrile and benzene selenol in reusable solvent polyethylene glycol (PEG-400) under ultrasonic irradiations (Scheme 47).^66^ A library of 2-amino selenopyridines with different substituents have been synthesized under this reaction conditions in moderate to good yields. *o*,*o*′-Disubstiuted aromatic aldehydes were also successful under the reaction conditions.

**Scheme 48:**
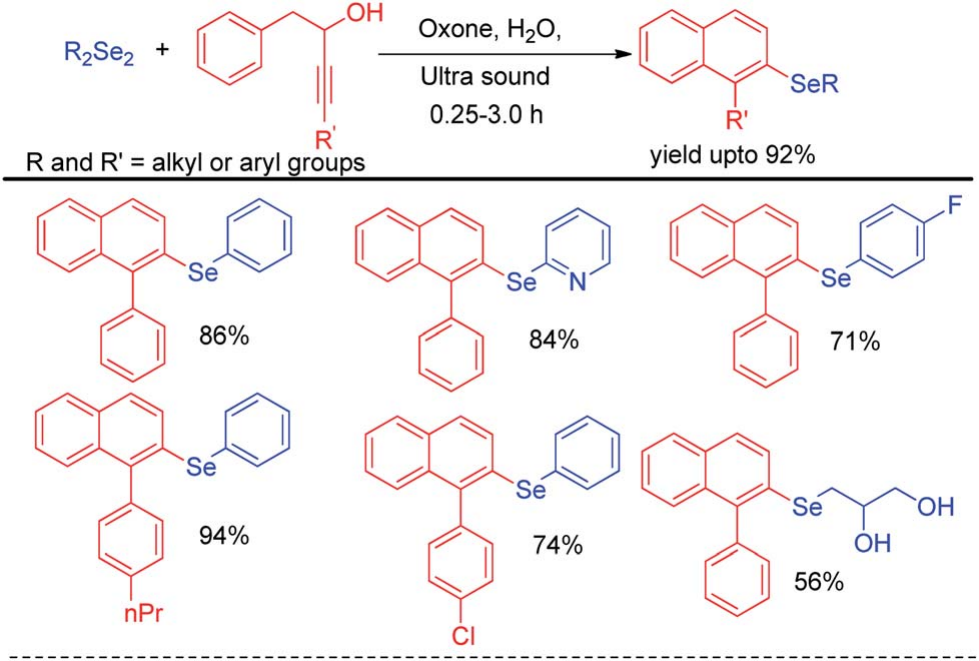
Ultrasound assisted synthesis of 2-organoselenyl-naphthalenes.

Perin *et al.* have developed ultrasound assisted synthesis of 2-organoselenyl-naphthalenes by reacting alkynoles with diaryldiselenides using oxone as oxidizing agent in water (Scheme 48).^67^ Electrophilic selenium species have been generated by the cleavage of Se–Se bond of diaryl selenides in the presence of oxone. Cyclization of alkynoles in presence of electrophilic selenium species led to the formation of product. Several substituted 2-organoselanyl-naphthalenes have been obtained in moderate to good yields in a short of time by this protocol. It was observed that the reaction requires 72 hours under conventional heating and thus the use of ultrasound for this reactions was time and energy efficient by large extent.

## 7 Under electrochemical cell

Electrochemistry has appeared as a sustainable alternative for organochalcogenide especially due to the current efficiency, high yield and control of the process.

**Scheme 49:**
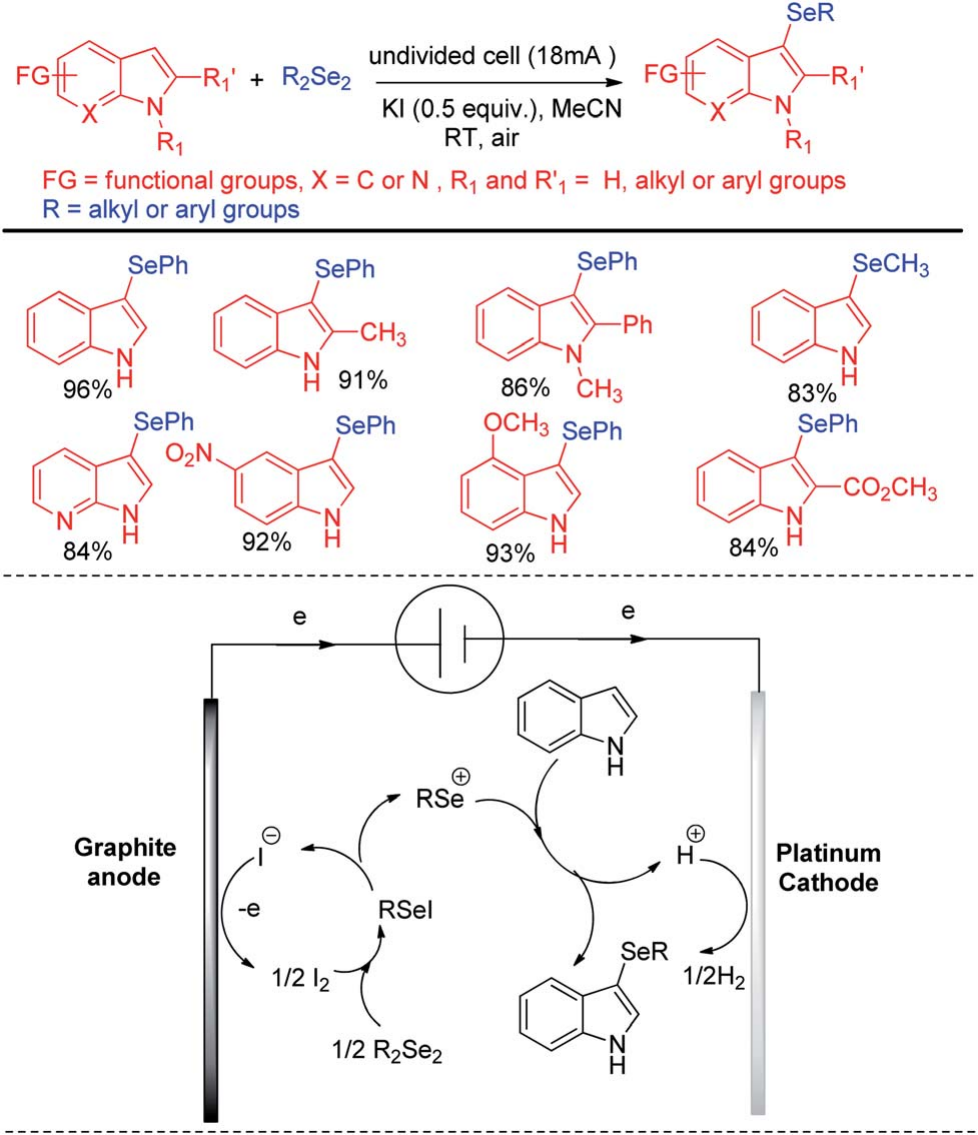
Iodide catalyzed electrochemical C–H selenylation of indoles.

**Scheme 50:**
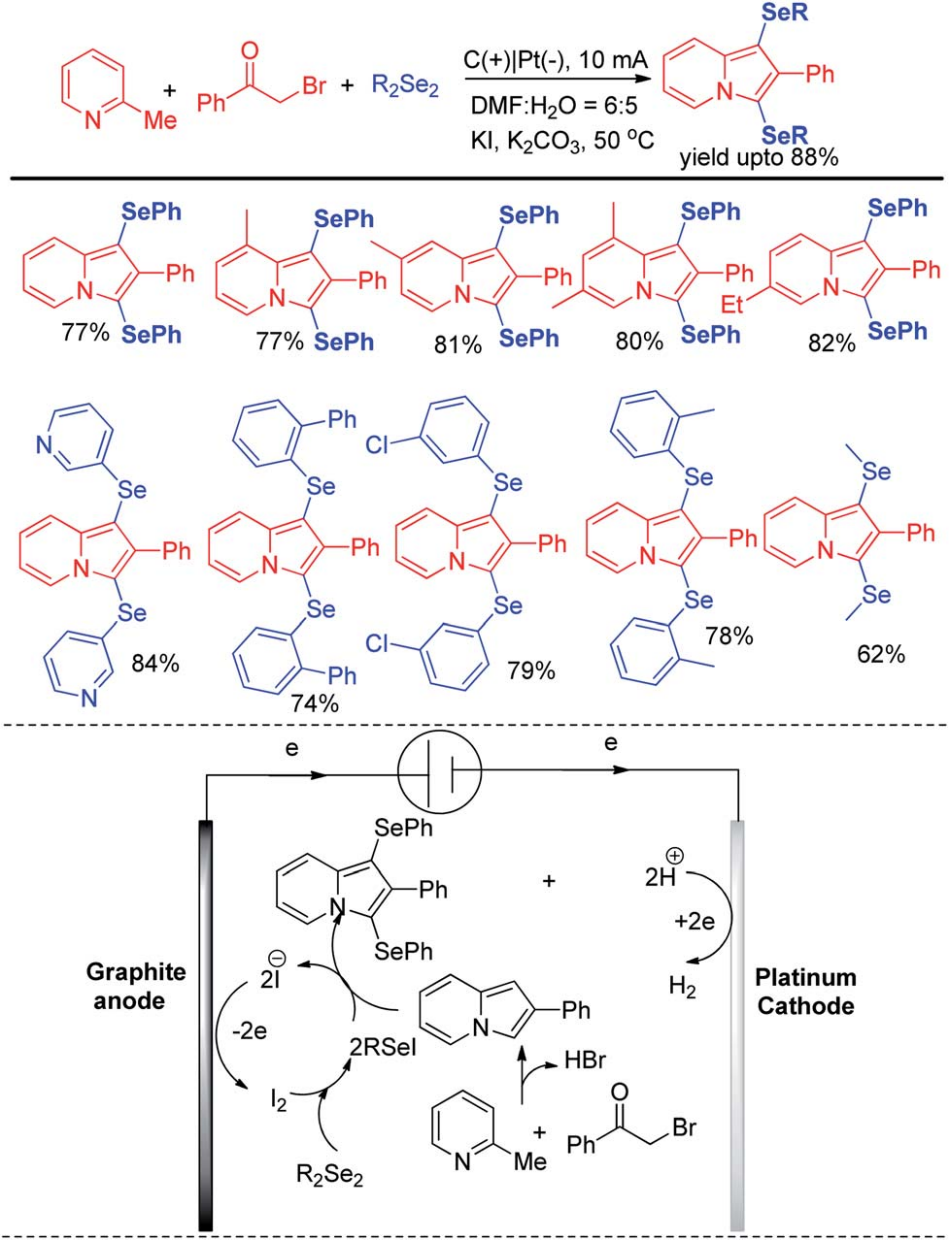
Synthesis of diselenylindolizines by multicomponent reaction under electrochemical cell.

Thus reactions can become economically and environmentally viable by using electrochemical procedures.^68^ Jian and Sun *et al*. reported iodide ion catalyzed electrochemical selenylation of indoles and other heteroarenes in absence of any oxidizing agent (Scheme 49).^69^ The authors performed the reactions under undivided cell with graphite anode and platinum cathode under constant current of 18 mA. The authors have proposed plausible path for this reaction bearing oxidation of iodide in anode and synthesis of active electrophile PhSeI. The product was obtained by usual deprotonation from heteroarenes where the liberated proton was reduced to hydrogen in cathode of the cell. Cao and Yu *et al.* reported an electrochemical cell based multicomponent reaction of 2-methyl pyridine, ketones and organodiselenides for the synthesis of diselenylated indolizines by using K_2_CO_3_ as base and KI as electrolyte under constant current of 10 mA (Scheme 50).^70^ The reaction required heating at 50 °C. Large array of substituents were well tolerated in pyridine ring and aryl rings of diaryldiselenides. The *in situ* formed indolizidine underwent reaction with in presence of iodide under electrochemical cell to form the product.

## 8 Future perspectives

In spite of tremendous importance of transition metal catalyst free protocols for the synthesis of aryl and heteroaryl selenides and tellurides challenges and opportunities remain: (1) C–Se bond formations in the electron deficient positions of arenes and heteroarenes. (3) Synthesis of aryl and heteroaryl tellurides is still less explored specially under metal free conditions. It is anticipated that these problems can be solved by developing proper organocatalytic systems in combination with photoredox catalysis.

## 9 Conclusions

Since last decade, a number of transition metal catalyst free protocols have been developed for C–Se and C–Te bond formations at the unsaturated carbon centres of aromatic rings and heterocycles. The advent of sustainable tools in performing C–Se & C–Te cross-coupling under transition metal catalyst free conditions has resulted tremendous advantages in the field of sustainable organic synthesis. Apart from conventional methods, reactions under sustainable energy sources *e.g.* visible light photocatalysis, microwave irradiation, ball milling, ultrasound for the synthesis of aryl and heteroaryl selenides and tellurides have been highlighted in this review. Beside aryl selenides, different important Seneo-substituted heterocycles such as indole, imidazopyridine, quinolone, 1,3,4-oxazole, imidazothiazole, naphthofuran, imidazopyrimidine, pyrazoles, imidazole, thiophene, isocoumarin have been synthesized under transition metal free conditions and are discussed along with the mechanisms. More importantly these methodologies can be applied for the synthesis of aryl and heteroaryl selenide based medicinal and pharmaceutically important molecules and natural product by sustainable paths.

## Conflicts of interest

There are no conflicts to declare.

## Supplementary Material
